# Electrochemical and Nanomaterial‐Based Strategies for Nonenzymatic Glucose Detection: A Review

**DOI:** 10.1002/open.202500304

**Published:** 2025-07-21

**Authors:** Reagan Aviha, Gymama Slaughter

**Affiliations:** ^1^ Center for Bioelectronics Old Dominion University Norfolk Virginia 23508 USA; ^2^ Department of Electrical and Computer Engineering Old Dominion University Norfolk Virginia 23508 USA

**Keywords:** glucose biosensors, nonenzymatic, smartphone‐integrated

## Abstract

Electrochemical glucose sensing technologies have undergone significant evolution, with continual advancements aimed at improving sensitivity, selectivity, and user convenience. This review systematically explores the development of emerging nonenzymatic glucose sensor designs. Nonenzymatic sensors are critically evaluated for their ability to overcome enzymatic limitations, leveraging novel materials and catalytic mechanisms. Additionally, the emergence of smartphone‐integrated glucose monitoring systems is highlighted as the fifth generation, representing a paradigm shift toward personalized, real‐time healthcare management. Emphasis is placed on the strategies employed to reduce the working electrode potential and enhance analytical performance. Key analytical metrics and real‐sample applicability are evaluated, and persistent challenges including reliability, biocompatibility, and calibration‐free operation are identified. Further, this review provides a critical perspective on the trajectory of electrochemical nonenzymatic glucose sensor technologies and outlines future directions toward the development of next‐generation platforms for continuous and noninvasive glucose monitoring.

## Graphical abstract

This review critically evaluates emerging nonenzymatic glucose sensing platforms, smartphone‐integrated devices, and fifth‐generation strategies for continuous noninvasive glucose monitoring.

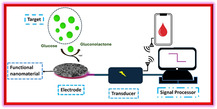

## 1 Introduction

**Figure 1:**
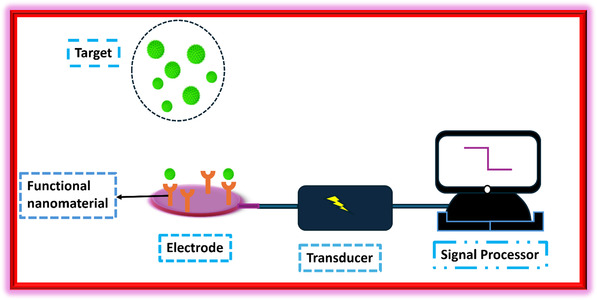
Schematic illustration of electrochemical sensor.

With the global diabetes burden projected to reach 700 million by 2045, the need for sensitive, reliable, and user‐friendly glucose monitoring technologies is increasingly urgent.^[^ ^1^, ^2^, ^3^, ^4^ ^]^ Electrochemical glucose sensors have played a critical role in enabling real‐time, decentralized monitoring due to their sensitivity, portability, and ease of integration with electronic devices.^[^ ^5^, ^6^, ^7^, ^8^ ^]^ These platforms typically consist of a sensitive element for glucose detection, a transducer to convert the biochemical signal into an electrical signal, and a processor for signal amplification and readout as illustrated in **Figure** **1**.

These sensors are broadly categorized into enzymatic and nonenzymatic systems, each with distinct mechanisms, advantages, and limitations. While first‐ to third‐generation enzymatic sensors have dominated the market, capitalizing on glucose oxidase or dehydrogenase for signal transduction, their dependency on biological recognition elements poses stability and reproducibility challenges.^[^ ^9^, ^10^ ^]^ In response, fourth‐generation nonenzymatic sensors have emerged, leveraging nanostructured catalytic materials to improve durability and simplify fabrication.^[^ ^11^ ^]^ Despite the extensive literature on nanomaterials for electrochemical sensing,^[^ ^12^, ^13^, ^14^ ^]^ prior reviews have primarily focused on material synthesis and sensor construction, offering limited critical evaluation of how these innovations translate to clinical or point‐of‐care (POC) utility.

This review departs from conventional surveys by systematically analyzing nonenzymatic glucose sensors and their integration in smartphone and wearable platforms, emphasizing design strategies aimed at lowering working potentials, enhancing analytical performance, and enabling calibration‐free, noninvasive detection. It highlights the transition toward personalized and real‐time monitoring through digital health integration, identifies persistent bottlenecks in selectivity, stability, and sample matrix interference, and outlines future research directions to bridge the gap between laboratory performance and clinical applicability.

## 2 Electrochemical Characterization Techniques

Electrochemical techniques play an important role in evaluating and optimizing glucose sensors by providing insight into reaction kinetics, interfacial electron transfer dynamics, and overall sensor performance. These methods enable real‐time detection, quantification of glucose concentration, and validation of electrode modification strategies, particularly for nanostructured and functionalized surfaces. Among the most widely applied techniques are cyclic voltammetry (CV), chronoamperometry (CA), electrochemical impedance spectroscopy (EIS), and potentiometry. Each technique serves a distinct purpose and offers complementary information critical for sensor design, calibration, and application‐specific deployment.

### 2.1 CV

CV is the most common technique used to investigate the redox behavior of modified electrodes and the electrocatalytic response to glucose. In a typical CV experiment, a triangular waveform is applied across a predefined potential window, and the resulting current is recorded as a function of the applied potential. The shape, peak current, and peak position of the resulting voltammogram provide valuable information about the reversibility, catalytic activity, and electrochemical stability of the sensing interface.^[^ ^15^ ^]^

**Figure 2:**
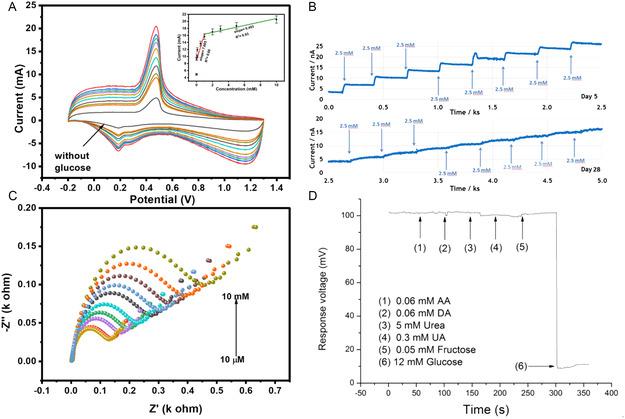
Electrochemical characterization of glucose sensor using A) cyclic voltammetry (CV), ^[^ ^17^ ^]^ B) chronoamperometry (CA) [Inset: linear curve], ^[^ ^18^ ^]^ C) electrical impedance spectroscopy (EIS),^[^ ^17^ ^]^ and D) potentiometry.^[^ ^19^ ^]^ Adapted with copyright permission from MDPI and IEEE.

In nonenzymatic glucose sensors, where the oxidation of glucose occurs directly at the electrode surface, the current response is linearly proportional to glucose concentration as shown in **Figure** **2**A. Thus, CV is used to establish the electrochemical mechanism of glucose oxidation, assess surface reaction kinetics, and determine the active surface area of nanomaterial‐modified electrodes. For example, CV has been employed to monitor oxidation peaks corresponding to glucose and to validate the electrocatalytic enhancement imparted by materials such as platinum (Pt), gold (Au), copper oxide (CuO), or doped carbon nanostructures. Moreover, CV is often combined with advanced techniques like differential pulse voltammetry (DPV) and square wave voltammetry (SWV) for enhanced sensitivity, particularly in low‐concentration regimes where Faradaic currents are minimal. Beyond sensing performance, CV also provides key insights into charge storage behavior and surface fouling, making it invaluable for evaluating sensor longevity and reusability.

### 2.2 CA

CA involves applying a constant potential to the working electrode and monitoring the resulting current as a function of time. This method is particularly well‐suited for real‐time glucose monitoring applications due to its operational simplicity and temporal resolution. As shown in Figure 2B, upon stepwise addition of glucose, the system exhibits a corresponding increase in current reflecting the catalytic oxidation of glucose at the electrode surface. Additionally, CA is frequently used to assess the dynamic response of sensors under steady‐state conditions and to determine parameters such as sensitivity, linear range, response time, and limit of detection (LOD). The shape of the current–time (*i–t*) curve reveals diffusion‐controlled kinetics and provides quantitative information on the mass transport behavior of glucose toward the electrode surface.^[^ ^16^, ^17^ ^]^ Advanced electrode designs with hierarchical porosity and high surface‐to‐volume ratios often show accelerated current responses and lower steady‐state currents that are indicative of high catalytic efficiency. In addition to sensitivity analysis, CA is employed in interference studies to evaluate the selectivity of glucose sensors in the presence of electroactive species like uric acid (UA), ascorbic acid (AA), and acteaminophen (AP), which are commonly found in physiological fluids. This technique is critical for validating the real‐world applicability of glucose biosensors, particularly in complex matrices such as sweat, saliva, or interstitial fluid.

### 2.3 EIS

EIS is a powerful nondestructive technique used to characterize the interfacial properties of electrodes, particularly those functionalized with nanomaterials or biorecognition elements. It involves applying a small sinusoidal voltage perturbation over a range of frequencies and measuring the resultant current to derive the impedance spectrum. The results are commonly presented as Nyquist plots (Figure 2C), with semicircles corresponding to charge transfer resistance (R_ct_) and straight lines indicating diffusion‐limited processes (Warburg impedance). The diameter of the semicircle is inversely related to the rate of electron transfer; hence, a decrease in R_ct_ following glucose addition signifies enhanced interfacial conductivity due to electrocatalysis.^[^ ^18^ ^]^ EIS is especially useful for tracking electrode modification steps and verifying the successful integration of nanomaterials onto the sensing interface. A well‐defined decrease in impedance upon glucose exposure can be directly attributed to catalytic activity, validating the electrode's functional design.

In sensors leveraging conductive polymers, metal organic frameworks (MOFs), or doped carbon‐based platforms, EIS helps quantify improvements in interfacial electron transfer, dielectric behavior, and double‐layer capacitance, which are critical to improving analytical performance. Furthermore, EIS is routinely used in long‐term stability studies to assess changes in electrode properties due to fouling, delamination, or degradation under physiological conditions.

### 2.4 Potentiometry

Potentiometric sensing is based on the measurement of potential differences between a working and reference electrode in the absence of net current flow. Although less commonly used for direct glucose oxidation sensing, potentiometry provides a simple, low‐power alternative suitable for miniaturized and wearable platforms. Changes in electrode potential are recorded as a function of glucose concentration (Figure 2D), typically mediated by ion‐selective membranes that modulate interfacial potential through catalytic or diffusion processes.^[^ ^19^ ^]^ Potentiometric glucose sensors have been integrated into flexible and implantable formats due to their minimal power requirements and compatibility with microfabricated electronics. However, their relatively low sensitivity compared to amperometric methods limits their applicability in trace‐level glucose detection unless coupled with amplification strategies.

Therefore, the selection and integration of electrochemical techniques are critical in the comprehensive characterization of glucose sensors. While CV and CA remain essential for assessing redox activity and dynamic response, EIS provides insight into interfacial charge transfer processes and electrode modification integrity. Potentiometry, though less prevalent, serves specialized roles in miniaturized and wearable applications. These techniques guide the design of electrode materials as well as enable performance benchmarking under physiologically relevant conditions.

## 3 Nonenzymatic Electrochemical Glucose Sensors

The limitations of enzymatic approaches have catalyzed a paradigm shift toward fourth‐generation nonenzymatic electrochemical glucose sensors, which exploit nanostructured materials with intrinsic electrocatalytic activity to mimic enzymatic function. Materials such as noble metal nanoparticles (e.g., Pt, Au, palladium (Pd)), metal oxides, transition metal alloys, conducting polymers, and carbon‐based nanostructures like carbon nanotubes (CNTs) and graphene have been demonstrated to offer significant advantages.^[^ ^11^, ^20^, ^21^, ^22^ ^]^ These include lower cost, simplified fabrication, enhanced stability, wider operational ranges, and improved resistance to environmental interferences.^[^ ^11^, ^23^ ^]^ These features make them particularly attractive for integration into wearable and CGM devices.

### 3.1 Nonenzymatic Electrochemical Glucose Sensing Mechanisms

Building on the generational evolution of electrochemical glucose sensors, it is essential to understand the underlying operational principles that govern nonenzymatic electrochemical glucose sensors, where the reliance on biological recognition elements is replaced by the intrinsic electrocatalytic properties of nanomaterials. These sensors operate predominantly in alkaline media, where hydroxide ions (OH^−^) promote glucose oxidation. Nanostructured materials such as nickel (Ni), copper (Cu), and their oxides exhibit enzyme‐mimicking activity, offering enhanced surface area, electron conductivity, and stability.^[^ ^24^, ^25^ ^]^ For example, Conway et al. investigated glucose electrooxidation on a nickel hydroxide (Ni(OH)_2_) anode, where the catalytic cycle involves the following reactions^[^ ^26^ ^]^

(1) $$Ni{\left(OH\right)}_{2}+OH^{-}\;\to \text{NiOOH}+H_{2}O+e^{-}$$

(2) $$NiOOH+Glucose\;\to \;Ni{\left(OH\right)}_{2}+\;Gluconolactone$$

Similarly, copper‐based systems undergo^[^ ^25^, ^27^ ^]^

(3) $$CuO+OH^{-}\;\to \;CuOOH+e^{-}$$

(4) $$Cu{\left(OH\right)}_{2}+\;OH^{-}\to CuOOH+H_{2}O+e^{-}$$

(5) $$CuOOH+Glucose\to Cu{\left(OH\right)}_{2}+\;Gluconolactone$$

**Figure 3:**
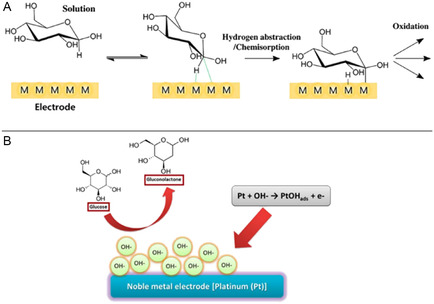
Schematic representation of A) chemisorption and B) IHOAM model of glucose oxidation.^[^ ^25^ ^]^ Adapted with copyright permission from MDPI.

Other transition metals and their oxides (e.g., Cobalt (Co), Manganese (Mn), and zinc (Zn)) follow analogous catalytic schemes, underlining the broad applicability of these materials. These nonenzymatic systems offer advantages such as improved reproducibility, low cost, and high operational stability. A schematic summarizing the mechanism of nonenzymatic glucose sensor is presented in **Figure** **3**. The electron transfer mechanisms in these systems are typically descried by two models: chemisorption and the incipient hydrous oxide adatom model (IHOAM).^[^ ^25^, ^27^ ^]^ In the chemisorption model illustrated in Figure 3A, glucose undergoes dehydrogenation upon adsorption, forming gluconolactone which subsequently hydrolyzes to gluconic acid. In contrast, the IHOAM model in Figure 3B emphasizes that the oxidation of glucose is mediated by hydroxylated metal sites (MOH_ads_), where metal oxides act as reactive intermediates under alkaline conditions, thereby promoting glucose oxidation.

The electrochemical behavior of transition metal‐based nanomaterials in glucose sensing is largely governed by their ability to exist in multiple oxidation states, enabling efficient redox cycling that facilitates the oxidation of glucose molecules. This redox flexibility is particularly pronounced under alkaline conditions, where hydroxide ions (OH^−^) in solution interact with metal ions at the electrode surface to form high‐valent species such as NiOOH or CuOOH that serve as the active intermediates in glucose oxidation.^[^ ^27^, ^28^, ^29^ ^]^ The transition from lower oxidation states (e.g., Ni^2+^, Cu^+^) to higher ones (e.g., Ni^3+^, Cu^2+^) under electrochemical polarization is a key mechanistic driver for catalytic activity. The general mechanism, as observed in materials like Ni, Cu, and extended to their oxide, sulfide, and nitride analogs, involves initial formation of metal hydroxide (M–OH) or oxide (MO), followed by electrochemical conversion into the active oxyhydroxide (MOOH) phase.^[^ ^28^ ^]^ Upon glucose adsorption, the MOOH species oxidize glucose into gluconolactone, concurrently regenerating the lower‐valent state and completing the catalytic cycle. These steps, illustrated in Figure 3B, emphasize the essential role of surface redox transitions and dynamic charge transfer in enabling sustained glucose oxidation.

### 3.2 Materials for Nonenzymatic Glucose Sensing

The design and functionality of nonenzymatic glucose sensors are deeply influenced by the physicochemical and electrochemical properties of the materials employed. Material morphology, encompassing surface area, porosity, particle size, and crystallinity significantly influences glucose sensing by modulating the density of electroactive sites, the efficiency of electron transfer, and the accessibility of the electrode surface to glucose molecules. Furthermore, crystallinity and defect density in these nanostructures influence the density of catalytic sites and the ease of electron hopping. Highly crystalline domains can stabilize redox‐active phases, whereas defect‐rich regions may act as active sites that lower the overpotential for glucose oxidation. Thus, nanocomposites that integrate transition metals with conductive scaffolds such as CNTs or graphene enhance electron transfer kinetics and mechanical stability while minimizing aggregation and biofouling.^[^ ^30^, ^31^ ^]^ Beyond these physical attributes, the underlying sensing mechanisms, including electron transfer pathways, redox transitions, and molecular adsorption dynamics, are equally critical in determining sensor sensitivity, selectivity, and stability under physiological conditions. Hence, material innovations have focused on improving electron transfer kinetics, enhancing biocompatibility, enabling miniaturization, and ensuring operational stability in complex biological matrices. This section offers a critical synthesis of four key material subclasses: noble metals, transition metal oxides and sulfides, carbon‐based nanomaterials, and conductive polymers, evaluating their structural attributes, electrocatalytic mechanisms, integration into biosensor platforms, and potential for next‐generation wearable and POC diagnostics.

#### 3.2.1 Noble Metal Nanomaterials: Catalytic Benchmarks for Nonenzymatic Platforms

**Figure 4:**
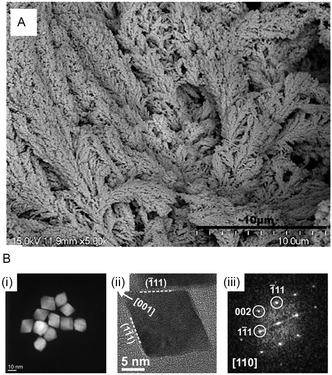
SEM image of A) gold microneedle electrode surface turned into extended branch‐like structures upon Pt‐black electrodeposition.^[^ ^35^ ^]^ B) High resolution transmission electron microscopy (HR‐TEM) images of 19 nm (i–iii) octahedral Pt NPs.^[^ ^36^ ^]^ Adapted with copyright permission from MDPI and Springer Nature.

Noble metals nanostructures such as Pt, Au, Pd, and silver (Ag) serve as reference catalysts due to their exceptional electrocatalytic efficiency, electrical conductivity, and biostability. Their d‐band electronic structures allow for facile adsorption and oxidation of glucose, particularly in alkaline media. Among these, Pt and Au remain dominant in nonenzymatic electrochemical sensors where enzyme‐free operation and long‐term stability are prioritized.^[^ ^32^, ^33^ ^]^ The morphology of noble metal nanostructures dramatically influences performance. Dendritic, flower‐like, and nanoporous morphologies significantly increase surface area, facilitating higher electroactive site densities. Recent advances in nanostructuring have dramatically enhanced surface‐to‐volume ratios and electroactive site densities, improving both sensitivity and detection range.^[^ ^34^ ^]^ These developments include mesoporous Pt electrodes designed for enzyme‐free detection in serum and whole blood. For example, Kim, Y. J. et al. introduced an innovative microneedle‐based sensor platform enhanced with porous Pt‐black coatings and wireless Bluetooth capability, demonstrating significant progress in both transdermal detection and real‐time signal delivery.^[^ ^35^ ^]^ The fabrication involved the electrochemical deposition of Pt‐black onto gold microneedle (AuMN) electrodes, creating a high‐surface‐area nanostructured interface that significantly enhances the electrochemical activity of the electrode. The porosity and branched‐like morphology of the Pt‐black layer depicted in the scanning electron microscopy (SEM) image in **Figure** **4**A play a critical role in amplifying the density of active sites for the direct oxidation of glucose at the Pt surface, facilitated by the IHOAM pathway. The sensor exhibited dual linear response ranges of 1–10 mM and 15–30 mM with respective sensitivities of 445.75 μA mM^−1^ cm^−2^ and 165 μA mM^−1^ cm^−2^. This dual‐range behavior is particularly advantageous for covering both hypoglycemic and hyperglycemic glucose concentrations, a performance characteristic rarely achieved by traditional enzymatic continuous glucose monitors (CGMs), which often suffer from limited linearity at extreme values. The reported LOD of 268 μM further confirms its capability for early detection within clinically relevant thresholds. From a performance optimization standpoint, the porous Pt‐black coating contributes to improved electrochemical signal amplification, low charge transfer resistance, and stable operation over extended wear periods.

Mazzotta et al. demonstrated the critical role of facet engineering in enhancing the electrocatalytic performance of noble metal nanomaterials for glucose sensing. They leverage facet‐controlled Pt nanocrystals with dominant {111} orientations, known to exhibit superior catalytic activity due to their high‐index surface planes to enhance catalytic turnover for glucose oxidation.^[^ ^36^ ^]^ These nanostructures were synthesized using a shape‐directed colloidal route, yielding octahedral Pt nanocrystals with high crystallinity and uniform morphology (Figure 4B). These octahedral Pt nanocrystals were subsequently immobilized onto conductive substrates to construct the working electrode of the sensor to exploit the DET between glucose and the Pt nanocrystal surface. The exposed {111} facets are known to promote adsorption and dehydrogenation of glucose molecules, accelerating their conversion to gluconolactone and gluconic acid. This facet‐dependent activity stems from the favorable electronic configuration and geometric arrangement of surface atoms, which lower the activation barrier for glucose oxidation and enhance turnover frequency. Consequently, the nanocrystal morphology not only increases the density of electroactive sites but also improves the reaction kinetics, resulting in a higher sensitivity and broader detection range.

Electrochemical evaluation in phosphate‐buffered saline (PBS) revealed a dual linear response region spanning 0.36–3.0 mM and 3.0–17 mM. The wide linear range highlights the ability of the sensor to maintain catalytic efficiency even at elevated glucose concentrations, a key limitation in many enzymatic platforms. Moreover, the nanocrystals demonstrated excellent signal stability, attributable to their highly crystalline surfaces, which resist oxidation and degradation under repeated cycling. By carefully balancing surface uniformity and nanocrystal dispersion aggregation was minimized. The nanozyme‐like behavior of the Pt {111}‐faceted structures also enhanced the robustness of the system, offering a viable alternative to enzymatic degradation and thermal instability commonly observed in biological sensors. However, despite these benefits, limitations such as high precursor cost, susceptibility to biofouling, and incomplete selectivity in complex fluids restrict their widespread clinical translation.

In contrast to these approaches, Katseli et al. introduced an innovative approach to nonenzymatic glucose sensing by designing a 3D‐printed, ring‐type electrochemical sensor specifically engineered for sweat‐based, noninvasive glucose monitoring.^[^ ^37^ ^]^ The sensor was fabricated using carbon‐based conductive filaments, shaped into a ring architecture via 3D printing, and subsequently modified with electrodeposited gold film. Gold nanostructures are known for their high resistance to poisoning by intermediate species and their favorable adsorption‐desorption kinetics, which facilitate efficient electron transfer. Glucose was oxidized via DET on the gold surface under alkaline conditions. The sensor exhibited a linear detection range of 12.5–400 μM and a low LOD of 1.2 μM, both of which align with physiological glucose levels found in eccrine sweat (typically 10–200 μM). This level of sensitivity is particularly significant given the challenges associated with nonblood matrices, where lower analyte concentrations and the presence of coexisting bioanalytes can diminish signal strength. Additionally, this low‐cost and customizable additive manufacturing technique enabled rapid prototyping and structural flexibility, providing a user‐centric platform compatible with wearable formats. The device was further designed to interface with a smartphone‐based potentiostat, thereby enabling decentralized, POC testing through portable and real‐time data acquisition.

Comparatively, Au‐based structures often outperform Pt due to their superior resistance to poisoning by reaction intermediates and their unique electronic structure as characterized by filled d‐orbitals and reduced surface adsorption of organic species.^[^ ^12^ ^]^ Varieties of Au nanostructures such as nanowires, nanotube arrays, nanoporous films, and 3D electrodes have shown promise in achieving high electrocatalytic response and sensitivity.^[^ ^31^, ^38^, ^39^ ^]^ However, limitations persist including high cost, potential selectivity issues in complex biological matrices, and susceptibility to interference by competing analytes.

#### 3.2.2 Transition Metal‐Based Nanostructures: Tunable Electrocatalysts

To overcome the limitations of noble metals, transition metal‐based materials have garnered interest due to their low cost, good electrocatalytic behavior, and tunable redox properties. Ni, Cu, Co, Mn, Zn, and iron (Fe) along with their oxides and sulfides are widely studied.^[^ ^27^, ^29^, ^40^, ^41^, ^42^ ^]^ NiO, for example, has demonstrated enhanced glucose oxidation kinetics compared to pure Ni, attributed to its surface hydroxylation in alkaline media. Cu‐based materials, particularly CuO and Cu_2_O, have enabled nonenzymatic platforms with superior sensitivity. Chen et al. reported a highly sensitive nonenzymatic glucose sensor based on hierarchical CuO nanostructures, achieving an impressive sensitivity of 1.18 mA mM^−1^ cm^−2^ and an extended linear range up to 5.53 mM.^[^ ^42^ ^]^ The sensor's remarkable performance is attributed to the deliberate engineering of its hierarchical morphology, which significantly enhanced the electroactive surface area and facilitated improved electron transfer kinetics.

**Figure 5:**
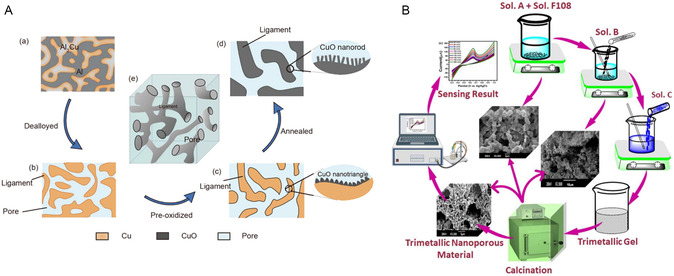
Schematic of the preparation and microstructural evolution: A) a) as‐spun Al–33.3 wt% Cu ribbon, b) dealloyed porous structure, c) pre‐oxidized Cu/CuO ligaments, d) annealed CuO nanostructure, and e) 3D view of the annealed sample.^[^ ^42^ ^]^ B) Modified glassy carbon electrodes were prepared by drop‐casting separately synthesized CuO, CuO/Ag, or CuO/Ag/NiO dispersions. After drying at room temperature, electrodes were electrochemically activated via CV (–1.0 to +2.0 V) in 0.1 m HNO_3_ until a stable response was observed.^[^ ^44^ ^]^ Adapted with copyright permission from Springer and Nature.

The CuO nanostructures were synthesized via a roller melt spinning technique followed by thermal annealing, resulting in firecracker‐shaped nanorod architectures with 3D interconnected nanopore (**Figure** **5**A). This structural configuration not only increased the density of catalytic active sites but also promoted efficient mass transport and analyte accessibility, both critical factors for high‐performance glucose sensing. The glucose oxidation mechanism on CuO surfaces primarily relies on redox cycling between Cu(II) and Cu(III) species in alkaline media (Equation (3), (4), (5), (6)). The hierarchical design is particularly beneficial for mitigating diffusion limitations and enabling rapid charge transfer, thereby improving both sensitivity and response time. To optimize performance, synthesis parameters such as pre‐oxidation time and washing cycles were controlled to tailor the morphology and crystallinity of the CuO nanostructures. Electrochemical characterization, including CV and CA, confirmed the high responsiveness of the sensor. Additionally, the sensor demonstrated good anti‐interference behavior against common physiological species such as AA and UA, suggesting strong potential for use in complex biological samples.

In addition to copper‐based systems, cobalt oxides (e.g., Co_3_O_4_ and CoOOH) have also demonstrated promising glucose sensing performance under similar alkaline conditions.^[^ ^43^ ^]^ These materials operate through analogous redox mechanisms involving the transition of Co(II) to Co(III) or Co(IV) states, which participate in glucose oxidation via high‐valent intermediates. Both copper and cobalt‐based systems offer significant advantages in terms of material abundance, cost‐effectiveness, and electrocatalytic activity, positioning them as viable alternatives to noble metal‐based sensors. Naikoo et al. introduced a highly sensitive ternary composite of CuO/Ag/NiO, demonstrating exceptional performance with a sensitivity of 2895.3 μA mM^−1^ cm^−2^ and a LOD of 0.1 μM across a linear range from 0.001 to 5.50 mM.^[^ ^44^ ^]^ The sensor's outstanding analytical performance is attributed to the synergistic integration of the three transition metal components, each contributing distinct electrochemical properties that collectively enhance electron transfer kinetics and catalytic site density. CuO, CuO/Ag, and CuO/Ag/NiO nanocomposites were synthesized separately and drop‐casted on the surface of glassy carbon electrode (GCE) (Figure 5B). The ternary composition allowed for the tuning of surface morphology and optimization of the redox‐active interface. SEM and X‐ray diffraction (XRD) analyses confirmed the formation of well‐dispersed nanostructures with interconnected porosity, which facilitated rapid glucose diffusion and increased electrode–analyte interactions.

The glucose oxidation process on the CuO/Ag/NiO electrode is governed by multi‐step redox reactions, where the transition metals cycle between their low and high oxidation states in alkaline medium. Specifically, Cu(II) and Ni(II) are electrochemically oxidized to Cu(III) (as CuOOH) and Ni(III) (as NiOOH), respectively, both of which serve as active sites for glucose oxidation. Ag nanoparticles, known for their high electrical conductivity and electron shuttling properties, enhance charge transfer at the electrode–electrolyte interface, reduce overpotential, and stabilize the composite architecture by preventing agglomeration of the metal oxide phases, further improving the response speed and sensitivity. The resulting material exhibits a high electroactive surface area and abundant catalytic sites, both of which are essential for achieving ultrasensitive glucose detection, especially at low concentrations relevant for noninvasive biofluids such as sweat or saliva. CV and CA demonstrated the reproducibility and stability of the sensor under controlled laboratory conditions. While promising for real‐time applications, questions remain regarding the long‐term electrochemical stability and biocompatibility of such complex composite systems, particularly in wearable or implantable devices.

#### 3.2.3 Carbon‐Based Nanomaterials: Conductive Scaffolds with Tunable Surface Chemistry

Carbon nanostructures such as graphene derivatives, CNTs, carbon dots, and carbon fibers offer distinctive mechanical and electrical properties beneficial to sensor applications. Their high aspect ratio, chemical stability, and inherent conductivity facilitate efficient electron transfer, and signal transduction.^[^ ^45^ ^]^ A defining advantage of carbon‐based systems lies in their surface modifiability, especially through heteroatom doping. The substitution of carbon atoms with nitrogen (N), boron (B), sulfur (S), or phosphorous introduces localized charge delocalization, defect sites, and catalytic hot spots. For example, nitrogen doping increases the electron‐donating ability and creates pyridinic and graphitic nitrogen species that enhance catalytic interactions with glucose molecules.^[^ ^46^ ^]^ These heteroatoms not only improve electrocatalytic activity but also boost sensor sensitivity and selectivity in the presence of physiological interferents like AA or UA.

**Figure 6:**
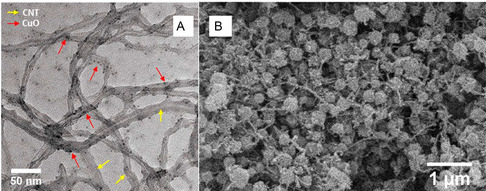
TEM image of A) the prepared CNTs/CuO NC. SEM images^[^ ^48^ ^]^ and B) the Ag@ZIF‐67/MWCNT.^[^ ^49^ ^]^ Adapted with copyright permission from MDPI and Elsevier.

Additionally, carbon nanomaterials serve as platforms for nanocomposite formation with metal or metal oxide nanoparticles, enhancing overall sensor performance through synergistic effects.^[^ ^47^ ^]^ Geetha et al. developed a nonenzymatic electrochemical glucose sensor utilizing CNT/CuO nanocomposite platform, specifically designed for glucose detection in sweat.^[^ ^48^ ^]^ The integration of CNTs and copper materials is known to enhance both the electrocatalytic response and conductivity, offering an effective pathway for noninvasive biosensing in biofluids like sweat. The sensor exhibited a sensitivity of 15.0 mA mM^−1^ cm^−2^ across a narrow but physiologically relevant range of 5–100 μM and a LOD of 3.90 μM. The fabrication strategy involved a simple wet‐chemical synthesis approach followed by drop‐casting on GCE to produce well‐integrated CNT/CuO nanocomposites with enhanced interfacial contact. Transmission electron microscopy (TEM) (**Figure** **6**A) and SEM analyses confirmed the distribution of CuO nanoparticles along the CNT framework, resulting in a hierarchically porous network with a high surface area and accessible electroactive sites.

The glucose sensing behavior is governed by the redox activity of Cu species, which undergo reversible transformation between Cu(II) and Cu(III) oxidation states in alkaline media, according to the IHOAM. This catalytic cycle is facilitated by the high surface energy of nanoscale CuO and accelerated by the rapid electron transfer kinetics enabled by the CNT backbone. The high aspect ratio and conductive sp^2^‐carbon network of the incorporated CNTs significantly reduce interfacial resistance, promote rapid charge mobility, and stabilize the nanocomposite under continuous electrochemical cycling. However, the limited dynamic range may constrain broader applicability across varying glucose concentrations in complex biological environments.

CNT‐based composites continue to play a transformative role in the design of high‐performance electrochemical glucose sensors due to their exceptional electrical conductivity, high surface area, and ability to facilitate electron transfer. Building on this, Elizbit et al. synthesized a porous zeolitic imidazolate framework (ZIF‐67) integrated with MWCNTs and decorated with AgNPs to create a hybrid sensor matrix.^[^ ^49^ ^]^ The architecture was strategically designed to exploit the individual strengths of each constituent material: porosity and high surface area from ZIF‐67, excellent electrical conductivity and mechanical stability from MWCNTs, and strong electrocatalytic activity from AgNPs. This multifunctional composite shown in Figure 6B demonstrated a sensitivity of 13.014 μA μM^−1^ cm^−2^ within a narrower linear range of 33–400 μM and an improved LOD of 0.49 μM. The synergistic integration of conductive MWCNT with AgNP‐enhanced electrocatalysis offers a promising route for improving target accessibility and signal amplification.

The fabrication of the sensor involved the synthesis of ZIF‐67 crystals followed by their physical integration with MWCNTs to create a conductive network. ZIF‐67, despite being a relatively fragile MOF in aqueous conditions, benefits from the protective CNT framework, which limits its degradation while preserving its porosity and enhancing analyte pre‐concentration effects. The MWCNTs served not only to improve electron transfer but also to provide mechanical robustness and structural connectivity within the porous matrix. Subsequent decoration with AgNPs was achieved via in‐situ chemical reduction, allowing uniform dispersion of catalytically active sites across the composite. The sensor operates via the direct electrooxidation of glucose on the surface of Co(II) in ZIF‐67. The Co(II) serves as primary catalytic sites where glucose is adsorbed and oxidized to gluconolactone, releasing electrons that are rapidly transferred through the MWCNT network to the electrode substrate.

Advancing toward wearable sensor formats, Xia et al. developed a flexible electrochemical glucose sensor by embedding a bimetallic Ni‐Co MOF within a CNTs/MWCNTs/PDMS (polydimethylsiloxane) matrix (CMP) using a dual‐stamping method, enabling precise, scalable deposition of functional layers onto flexible substrates.^[^ ^23^ ^]^ The Ni‐Co modified CMP (NCMP) device achieved a sensitivity suitable for physiological glucose detection (6.78 μM LOD) and a dynamic linear detection range of 20 μM to 1.1 mM. The fabrication process involved the synthesis of the Ni–Co MOF via a solvothermal method, followed by its uniform dispersion within a CNT ink. This composite was then transferred onto a PDMS film through a dual‐stamping procedure, which allowed sequential printing of the active material and electrode patterns while preserving material integrity and flexibility. The CMP platforms offer a robust electrode platform, the introduction of hierarchical porosity, metallic sites, and flexible substrates is proving crucial in tailoring sensors for real‐world biofluid monitoring applications. The dual‐metal MOF structure offered several performance advantages: the combination of Ni and Co in the MOF lattice introduced synergistic redox activity, creating abundant electrocatalytic sites that enhanced glucose oxidation efficiency. In alkaline media, Ni and Co species undergo redox transitions (e.g., Ni^2+^/Ni^3+^, Co^2+^/Co^3+^) that facilitate the indirect oxidation of glucose to gluconolactone via formation of high‐valent oxyhydroxide intermediates (NiOOH, CoOOH), improving current response and signal stability. However, the complexity of synthesis and potential reproducibility issues in fabrication remain technical barriers for mass production and clinical adoption.

#### 3.2.4 Conductive Polymers

Conductive polymers such as polyaniline (PANI), polypyrrole (PPy), polythiophene (PTh), polyacetylene, and PEDOT (Poly(3,4‐ethylenedioxythiophene) possess conjugated backbones that support delocalized electron transport. Their flexibility in film formation, mechanical stability, and ability to host dopants make them attractive for sensor fabrication.^[^ ^23^, ^50^ ^]^ Pal et al. developed a PANI‐AgNPs hybrid sensor capable of simultaneously detecting glucose and urea, achieving a low LOD of 1.9 μM.^[^ ^51^ ^]^ The incorporation of AgNPs not only enhanced electron transfer kinetics but also imparted catalytic activity toward glucose oxidation in alkaline conditions, illustrating a dual‐functional sensing interface. In this redox‐mediated process, Ag^+^ species are regenerated through anodic polarization, maintaining sustained catalytic turnover. The conjugated π‐electron backbone of PANI promotes rapid electron transport along the polymer chains, enhancing current response and lowering overpotential for glucose oxidation.

**Figure 7:**
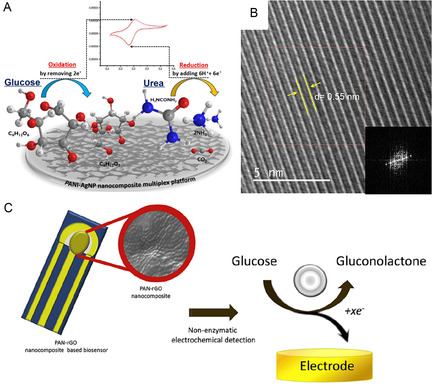
The schematic representation of A) PANI‐AgNP nanocomposite multiplex platform.^[^ ^52^ ^]^ B) TEM images of the Borophene nanosheet.^[^ ^52^ ^]^ C) PAN‐rGO nanocomposite‐based sensor proposed sensing mechanism.^[^ ^53^ ^]^ Adapted with copyright permission from Springer.

Expanding on this, Taşaltın et al. synthesized a novel PANI:B_12_ borophene composite, demonstrating an even higher sensitivity of 96.93 μA mM^−1^ cm^−2^ over the range of 1–12 mM.^[^ ^52^ ^]^ The use of borophene, a 2D material with high electron mobility, further highlights the strategic inclusion of emerging 2D nanomaterials to improve signal transduction in nonenzymatic formats. The fabrication process involved the mixing of PANI in the presence of exfoliated β_12_ borophene sheets, facilitating uniform dispersion of the 2D material within the PANI matrix. The conductive framework of the PANI backbone maximized interfacial contact between the polymer and borophene, which is critical for optimizing electron transfer kinetics. The inclusion of β_12_ borophene introduces a high density of delocalized π‐electrons and low energy barriers for charge transport, which synergistically enhance the overall electrocatalytic activity of the composite. The glucose oxidation on the PANI:borophene interface proceeds via a DET pathway in alkaline media. The borophene layers (**Figure** **7**A) provide abundant electroactive sites that facilitate the adsorption and dehydrogenation of glucose molecules, forming gluconolactone as the main oxidation product. Morphological analyses using SEM and TEM confirmed the uniform distribution of borophene within the PANI matrix and the formation of interconnected conductive networks, which are crucial for maintaining sensor stability and reproducibility.

Karakuş et al. reported a polyacrylonitrile‐reduced graphene oxide (PAN‐rGO) composite sensor, which operated effectively across 0.75–12 mM and reached a sensitivity of 49 μA mM^−1^ cm^−2^ with a LOD of 0.6 mM.^[^ ^53^ ^]^ While its sensitivity was modest relative to borophene‐based or AgNP‐enhanced systems, the simple synthesis route and mechanical stability of the PAN‐rGO matrix suggest suitability for scalable and low‐cost sensor fabrication. The fabrication process involved the chemical reduction of GO, followed by mixture of PAN:rGO (1:1 mass ratio). The PAN‐rGO nanocomposite was drop‐casted on a gold electrode substrate. The resulting nanocomposite exhibited a highly porous morphology with interconnected conductive pathways, as confirmed by SEM imaging (Figure 7B), which is critical for facilitating glucose diffusion and electron transport. The sensing process involves the adsorption of glucose molecules onto defect‐rich regions of the rGO sheets, where residual oxygen‐containing groups and structural irregularities serve as catalytic sites. Upon adsorption, glucose undergoes dehydrogenation to form gluconolactone, with concomitant electron release that contributes to the electrochemical signal. This mechanism, illustrated in Figure 7C, underscores the synergy between PAN's electron‐conducting network and rGO's catalytic interface, which together facilitate robust glucose oxidation under ambient conditions.

The judicious selection of nanomaterials in glucose sensor development is a multi‐factorial decision, driven by considerations of electrochemical performance, mechanical and chemical stability, biocompatibility, scalability, and integration capability with digital health platforms. There is a clear trend toward leveraging the intrinsic conductivity, electrocatalytic properties, and structural tunability of conducting polymers and graphene derivatives. Nonenzymatic platforms demonstrate increasing competitiveness, offering lower LODs and simpler fabrication routes. Noble metals offer benchmark catalytic activity but are constrained by cost and selectivity issues. Transition metals and their oxides provide tunable and cost‐effective alternatives, especially when configured as multi‐metallic composites. Carbon‐based materials, especially when heteroatom‐doped emerge as the most versatile class, enabling nonenzymatic sensing. Conductive polymers add value by offering mechanically adaptive matrices for wearable bioelectronics.

Moving forward, the design of polymer/nanomaterial hybrids with tailored porosity, surface functionality, and biocompatibility will be essential to bridge the performance gap in nonenzymatic sensing performance and system integration, including wireless data transmission, closed‐loop therapeutic feedback, and long‐term biointerface stability, ultimately advancing sensor platforms for POC and wearable healthcare applications.

#### 3.2.5 Integration of Smart Technologies in Glucose Sensors

The emergence of fifth‐generation glucose sensors represents a paradigm shift in biosensing, marking a transition from traditional, static measurements to dynamic, intelligent, and user‐centered systems. These platforms are characterized by their integration with smartphones, wireless data transmission modules, and algorithmic analytics that collectively support real‐time, minimally invasive glucose monitoring and autonomous glycemic control. This evolution is primarily driven by the need to improve patient compliance, enable early intervention, and support personalized disease management through seamless integration with daily life.

Smartphones, equipped with high‐performance processors (e.g., Apple A‐series, Qualcomm Snapdragon), now function as portable processing hubs that interface directly with biosensors. Their computational capabilities facilitate real‐time signal acquisition, advanced electrochemical data interpretation, and remote diagnostics through cloud‐based infrastructures.^[^ ^5^, ^54^, ^55^, ^56^, ^57^ ^]^ This enhances usability, expands clinical reach, and allows for round‐the‐clock monitoring with instant feedback. Mobile applications serve as intuitive user interfaces, offering visualizations of glucose trends, predictive analytics, and automated alerts. These capabilities are further enhanced by artificial intelligence and machine learning algorithms, such as support vector machines, convolutional neural networks, and decision trees, which can forecast glycemic fluctuations and classify risk profiles with greater precision than conventional methods.^[^ ^57^ ^]^

Wireless communication protocols underpin the functionality of these smart sensing ecosystems. Bluetooth remains the most widely adopted due to its universal compatibility and moderate energy demand. It operates within the 2.402–2.480 GHz range and supports continuous data transmission. However, concerns related to electromagnetic interference, data latency, and cybersecurity require further innovation in signal shielding and encryption. Near‐field communication, while operating at 13.56 MHz and limited to short‐range interactions, offers advantages in low‐power operations and quick, user‐initiated data transfers. Infrared communication is also used in some closed‐loop wearable systems, offering precise point‐to‐point connectivity; yet, its dependence online‐of‐sight and reduced compatibility with opaque or curved surfaces restricts its broader application. Emerging alternatives such as ZigBee and Wi‐Fi offer opportunities for mesh networking and wider area connectivity, though often at the expense of higher power consumption.^[^ ^58^, ^59^ ^]^

The integration of these communication technologies enables a new class of wearable and portable biosensing devices that go beyond passive monitoring. For instance, smartphone‐linked biosensors can automatically calibrate in response to environmental or physiological fluctuations, provide real‐time decision support to patients and clinicians, and enable remote monitoring in telemedicine contexts. Additionally, many platforms now support bidirectional data flow, allowing physician‐directed adjustments or behavioral prompts through mobile applications. This connectivity establishes a digital feedback loop that enhances adherence, minimizes clinical errors, and supports behavioral interventions. Despite these advances, several challenges must be addressed to ensure the clinical utility and consumer adoption of such platforms. Power consumption in continuous monitoring systems remains a bottleneck, particularly in battery‐constrained wearables. Future designs must prioritize energy‐efficient materials, energy harvesting mechanisms (e.g., triboelectric or enzymatic biofuel cells), and adaptive sampling rates to extend operational life. Furthermore, data privacy and interoperability with existing electronic health records demand secure cloud architecture and regulatory harmonization. User interface design must also consider accessibility across diverse demographics, including older adults or patients with impaired vision or dexterity.

**Figure 8:**
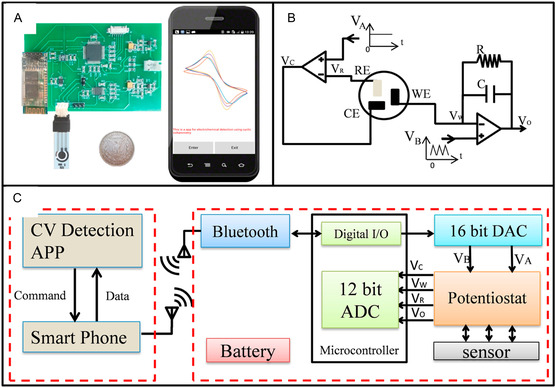
Smartphone‐based portable glucose monitoring. A) A smartphone‐based glucose detection system with a modified glucose electrode, a portable electrochemical circuit board, and a smartphone. B) Schematic diagram showing the modification of the glucose electrode. C) Schematic diagram of the smartphone‐based glucose monitoring system.^[^ ^60^ ^]^ Adapted with copyright permission from Elsevier.

The fusion of glucose biosensing with internet‐of‐things (IoT), wireless communication, and AI represents a transformative leap toward autonomous, patient‐specific, and clinician‐assisted glycemic management. By embedding electrochemical sensors into connected health platforms, future devices are expected to deliver not only precision diagnostics but also contextual health insights. These developments foreshadow a decentralized, real‐time healthcare model where glucose management becomes predictive, preventative, and participatory, as illustrated in **Figure** **8**.^[^ ^60^ ^]^ Improvements in sensor miniaturization, biocompatibility, and response latency are paving the way for sixth and seventh‐generation devices, which may combine nanobiohybrid materials, artificial intelligence, and closed‐loop insulin delivery systems to achieve autonomous glycemic control.

## 4 Challenges and Materials‐Driven Strategies for Enhancing Electrochemical Glucose Sensors

Electrochemical glucose sensors are evaluated across a set of standardized analytical metrics that determine their practical efficacy, especially in clinical or real‐world applications. These metrics include sensitivity, selectivity, linear detection range, response time, and LOD. Despite substantial advancements in glucose biosensing, numerous technical challenges remain, particularly in achieving high selectivity, sensitivity, and stability in complex biological environments. Addressing these limitations is vital for developing clinically reliable, long‐term glucose monitoring systems suitable for real‐world applications.

### 4.1 Selectivity and Sensitivity: Material Innovations

Achieving high selectivity and sensitivity is a primary challenge in electrochemical glucose sensing, especially in physiologically relevant matrices where interfering substances, such as AA, UA, and AP, coexist at concentrations comparable to or higher than glucose. In such settings, signal overlap can compromise the sensor's accuracy and reliability.^[^ ^61^ ^]^ To improve sensitivity, nanostructured materials such as rGO and CNTs have been widely employed due to their superior electrical conductivity and large electroactive surface area. These properties facilitate rapid electron transfer, thereby enhancing the signal amplitude in response to glucose oxidation.

#### 4.1.1 Sensitivity

Sensitivity defines the sensor's ability to translate small variations in glucose concentration into measurable current output, typically expressed in μA mM^−1^ cm^−2^. It is extracted from the slope of a linear calibration curve (current vs. glucose concentration), normalized by the electrode's active surface area. Therefore, high sensitivity is crucial for detecting low glucose concentrations, particularly in early‐stage diabetes or continuous glucose monitoring (CGM) applications. Sensitivity depends strongly on the electrode surface area, electron transfer kinetics, and catalytic properties of the sensing materials.^[^ ^62^ ^]^ Feng et al. demonstrated Ag@TiO_2_@metal nanosheets attaining a sensitivity of 0.788 μA μM^−1^ cm^−2^ at a low applied potential of 0.2 V, highlighting the importance of nanoscale structuring in enhancing signal transduction.^[^ ^63^ ^]^ Pd‐based composites such as Pd‐H and PdAl exhibited even higher sensitivities, with values reaching 602.3 and 581.4 μA μM^−1^ cm^−2^, respectively, which further improved upon post‐synthetic modification to 6.467 and 602.4 μA μM^−1^ cm^−2^.^[^ ^64^ ^]^ Wang et al. developed a nanoflower‐like MoS_2_@CuCo_2_O_4_ heterostructure, where the synergistic interaction between the high surface area MoS_2_ nanosheets and the redox‐active CuCo_2_O_4_ significantly enhanced glucose oxidation kinetics.^[^ ^65^ ^]^ The resulting sensor demonstrated a sensitivity of 1.303 μA mM^−1^ cm^−2^ within a linear detection range of 0.5–393 μM, a low LOD of 0.5 μM, and a rapid response time of 2.1 s. These metrics reflect the sensor's ability to detect low glucose concentrations with high reliability, suitable for biofluids such as sweat or saliva where analyte concentrations are relatively low.

Expanding on this approach, Arunbalaji et al. fabricated a CuO flake/MoS_2_ nanosheet composite that leveraged the catalytic redox activity of CuO and the conductive, layered architecture of MoS_2._^[^ ^66^ ^]^ This hybrid system exhibited a high sensitivity of 1055 μA mM^−1^ cm^−2^ across a physiological range of 0.1–10 mM and, within a narrower range of 35–800 μM, achieved a LOD of 0.017 μM and a swift response time of 2 s. The performance gains can be attributed to the increased electroactive surface area and enhanced charge transfer facilitated by the intimate interface between CuO flakes and MoS_2_ layers. In another approach, Wu et al. introduced a novel architecture combining star‐shaped copper microcrystals (SCSMs) doped with the conductive polymer PEDOT and integrated with MWCNTs.^[^ ^67^ ^]^ This composite exhibited dual linear ranges (0.495–374 μM and 0.374–3.446 mM), effectively covering both trace and elevated glucose levels. The sensor achieved a low LOD of 0.04 μM and a rapid response time under 4 s. The multi‐scale architecture, featuring high‐curvature SCSMs for increased catalytic activity, PEDOT for enhanced conductivity, and MWCNTs for structural stability and electron transport, illustrates the importance of hierarchical design strategies in achieving high‐performance biosensing platforms. Moreover, the integration of nanostructured morphologies (e.g., nanoflowers, flakes, or star shapes) offers tunable surface chemistry and porosity, which can be engineered to optimize sensitivity, detection limits (DLs), and response times.

#### 4.1.2 Selectivity

Selectivity represents a critical parameter in the performance evaluation of electrochemical glucose sensors, particularly for nonenzymatic platforms operating in physiologically complex environments. It defines the sensor's ability to distinguish glucose from structurally or electrochemically similar species commonly present in biological fluids such as blood, sweat, saliva, and interstitial fluid. Given the coexistence of numerous electroactive molecules, rigorous interference testing is indispensable to demonstrate the practical applicability and reliability of the developed sensor systems.

Physiological fluids often contain a broad spectrum of potentially interfering species, including UA, AA, DA, AP, lactic acid, galactose, mannose, cysteine, glutathione, lactose, and various inorganic ions (Na^+^, K^+^, Ca^2+^, Cl^−^). Many of these compounds exhibit oxidation potentials that overlap with glucose or participate in redox reactions at the working electrode, leading to false‐positive signals or capacitive artifacts that degrade the electrochemical signal. Cysteine and glutathione, for example, are strong reducing agents whose electrochemical oxidation often mimics the glucose response due to similar reaction pathways, resulting in misleading current signals. Additionally, ions such as Na^+^ and Cl^−^ can alter the double‐layer capacitance or induce ionic screening effects, introducing signal drift or background noise.

**Figure 9:**
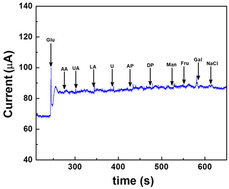
Selectivity of Au|Pt‐black|NF sensor towards glucose and common interferents under physiological conditions.^[^ ^35^ ^]^ Adapted with copyright permission from MDPI.

Selectivity remains a major challenge in nonenzymatic glucose sensing due to the presence of interfering species in physiological fluids.^[^ ^68^ ^]^ The Au|Pt‐black|NF sensor developed by Kim, Y. J. et al exhibit a working potential of +0.2 V vs. Ag/AgCl in PBS (pH 7.4), where the glucose signal amplitude significantly surpassed that of interfering species **Figure** **9**.^[^ ^35^ ^]^ Standard interference protocols involve spiking the test solution with interferents at concentrations 10‐fold higher than physiological levels and evaluating their effect on the current response, which illustrates a representative amperometric response curve, where the sensor demonstrates a sharp initial response to glucose and negligible current variation upon the sequential addition of interferents, thereby validating its high selectivity under simulated physiological conditions.

To further enhance molecular discrimination, surface engineering strategies involving MOFs and polymeric coatings have been widely adopted.^[^ ^69^, ^70^, ^71^ ^]^ MOFs offer tunable porosity, high surface area, and modifiable chemical environments, enabling size‐ and charge‐selective glucose recognition. Furthermore, biocompatible polymeric membranes, such as Nafion and chitosan, have been incorporated onto sensor surfaces to act as semi‐permeable barriers. These membranes permit glucose permeation while selectively excluding interfering analytes based on size or charge, thus improving the overall specificity of the sensor.^[^ ^70^, ^71^ ^]^ Liang et al. reported bimetallic MOF‐based sensors incorporating nickel–molybdenum (NiMo‐MOF) and cobalt–molybdenum (CoMo‐MOF) frameworks, both known for their redox activity and porosity.^[^ ^72^ ^]^ The NiMo‐ and CoMo‐MOF‐based sensors were functionalized with Nafion, a sulfonated tetrafluoroethylene ionomer known for its permselective properties. The sensor achieved a linear range up to15 mM with a sensitivity of 246.71 μA mM^−1^ cm^−2^, achieving a LOD of 26.17 μM, attributed in part to Nafion's role in excluding interfering analytes while enhancing ion conductivity. Another approach involves biopolymer‐based matrices. Chen et al. fabricated a dual‐range glucose sensor by integrating copper cysteamine complexes with chitosan on screen‐printed electrodes (SPEs).^[^ ^73^ ^]^ The biocompatible chitosan film served as both an immobilization scaffold and an interference barrier. The sensor displayed sensitivities of 588.28 μA mM^−1^ cm^−2^ (low range: 2.7 μM–1.3 mM) and 124.42 μA mM^−1^ cm^−2^ (high range: 1.3–7.7 mM). This dual linear range is especially useful for applications in precise glucose quantification across multiple biological matrices, including sweat and blood. This highlights the potential of polymer‐metal composites in achieving selective, tunable detection ranges suitable for diverse diagnostic contexts.

As nonenzymatic glucose sensors move toward clinical and POC applications, selectivity becomes as critical as sensitivity and stability. The development of functional interfaces that selectively transport glucose while rejecting interfering species remains a central challenge. Surface modification strategies such as combining nanostructuring, selective membranes, redox‐inactive supports, and machine learning‐assisted signal deconvolution can be implemented to further advance electrochemical sensor selectivity in complex, dynamic biological environments.

### 4.2 Addressing Analyte Adsorption and Nanoparticle Agglomeration

The adsorption of glucose and interfering species onto electrode surfaces, particularly within porous sensing matrices, can lead to reduced interaction between the active sites and the analyte. This adsorption, often attributed to high surface‐area‐to‐volume ratios in nanomaterial‐modified electrodes, results in signal attenuation and low sensitivity. To mitigate this, noble metal‐based sensors, especially those incorporating nanoparticles such as Au, Pt, and Cu have been explored as electrocatalytic enhancers and structural modifiers. These particles provide active sites for glucose oxidation and contribute to improved conductivity. However, excessive nanoparticle loading may induce agglomeration, which blocks active surfaces, restricts mass transfer, and compromises reproducibility and stability. Reproducibility reflects the consistency of the sensor's performance under identical testing conditions, either across multiple measurements with the same device or among devices fabricated under the same protocol. High reproducibility ensures reliability and minimizes calibration requirements, which is particularly valuable in scalable manufacturing. Therefore, controlled surface modification and the use of stabilizing agents (e.g., surfactants, polymers) are necessary to preserve nanoparticle dispersion while maximizing sensor performance.^[^ ^38^ ^]^

For example, electrodes fabricated via controlled electrodeposition or template‐assisted synthesis have shown relative standard deviations (RSDs) of less than 5% across repeated measurements or batches, satisfying regulatory expectations for clinical diagnostic devices.^[^ ^39^ ^]^ Eguilaz et al. enhanced sensor reproducibility and stability by functionalizing bamboo‐like CNTs with mercaptophenylboronic acid and decorating them with AuNPs.^[^ ^74^ ^]^ The resulting biocatalytic layer enabled reproducible electrochemical performance, with RSDs of 8.6% over 10 consecutive measurements on a single electrode and 4.5% across 10 different electrodes. The device retained stable activity for 14 days, highlighting the benefit of covalent functionalization and nanostructuring in improving repeatability and shelf‐life.

Stability denotes the sensor's ability to maintain its analytical performance over time, including during storage or prolonged use. It is typically quantified by measuring changes in sensitivity or baseline current over days or weeks of use. A stable sensor should retain at least 90% of its initial response after multiple cycles or extended storage under physiological conditions. The stability of nanostructured electrodes can be influenced by surface fouling, degradation of the sensing layer, or detachment of nanomaterials from the substrate. Strategies to enhance long‐term stability include surface passivation, polymer encapsulation, and covalent anchoring of nanostructures. Incorporating Nafion or Chitosan matrices has been shown to improve both environmental and operational stability without significantly compromising sensitivity.^[^ ^68^, ^70^, ^71^, ^72^, ^73^, ^75^ ^]^

### 4.3 Biocompatibility and Minimization of Biological Interference

In CGM applications, biocompatibility is a critical consideration. Exposure to biological matrices such as blood or interstitial fluid introduces risks of enzymatic degradation, immune response, and surface fouling by proteins or other macromolecules. To enhance compatibility, materials such as graphene derivatives, CNTs, and biocompatible polymers are employed to create inert, nontoxic surfaces that minimize adverse biological interactions. These materials can also inactivate or repel interfering biomolecules, thereby preserving sensor functionality over extended durations.^[^ ^76^, ^77^ ^]^

Polymer nanocomposites, particularly those incorporating carbon‐based materials, have gained increasing attention not only for sensing applications but also for broader roles in diabetes management, including drug delivery. As reviewed by Sen et al., carbon‐integrated polymer nanocomposites offer unique physicochemical properties such as high conductivity, flexibility, and biocompatibility.^[^ ^78^ ^]^ These multifunctional platforms can respond to glucose fluctuations with tunable release profiles, providing new therapeutic avenues for closed‐loop diabetes care systems. Similarly, Baruah et al. extensively discussed the biomedical potential of graphene‐based nanocomposites, including GO, rGO, GO quantum dots (QDs), and nano‐reduced graphene.^[^ ^79^ ^]^ These materials have shown promise in enhancing biocompatibility and surface functionalization. Their exceptional surface area and ease of chemical modification make them ideal candidates for next‐generation electrochemical nonenzymatic sensors.

Further, electrode degradation due to fouling or exposure to reactive intermediates poses another major limitation to sensor durability and repeatability. Fouling can result from the accumulation of proteins, salts, microorganisms, or oxidation by‐products on the electrode surface, ultimately inhibiting electron transfer and degrading sensor response. To combat fouling and electrode poisoning, antifouling agents such as noble metal nanoparticles (Au, Ag, Cu) are often incorporated into sensor designs. These materials resist biofouling and maintain electrode activity even in the presence of harsh electrolytes or biological by‐products, including chloride ions or reactive oxygen species. The development of self‐cleaning surfaces, anti‐biofouling coatings, and enzymatically inert interfaces continues to be an active area of research aimed at prolonging sensor lifespan and maintaining signal strength.^[^ ^80^ ^]^

Zhou et al. integrated GO nanohybrid structures into ZnOQDs deposited on steel substrates and demonstrated an improved corrosion‐resistant interface, suggesting a potential route for enhancing the long‐term operational stability of electrochemical sensors in harsh or biofluid environments.^[^ ^81^ ^]^ The protective characteristics of such nanostructured coatings are critical for extending sensor lifespan, particularly in wearable and implantable formats. Shang et al. further advanced the application of hybrid materials by designing a glucose biosensor based on Au/rGO integrated with Ti_3_C_2_ MXene. The resulting Au/rGO–Ti_3_C_2_ electrode exhibited a high sensitivity of 355 μA mM^−1^ cm^−2^ within a wide linear range of 10 μM to 21 mM and a low LOD of 3.1 μM.^[^ ^82^ ^]^ Importantly, this system displayed strong anti‐interference properties, with negligible signal perturbation from common electroactive species such as UA and AA, confirming its high selectivity and suitability for complex biological samples. An innovative approach introduced by González‐Martínez et al. focused on electrode surface modification by applying repeated mechanical wringing followed by electrochemical chronoamperometric treatment to generate roughened gold microstructures. This surface architecture significantly improved both sensitivity and antifouling characteristics during glucose detection in human serum.^[^ ^83^ ^]^ Enhanced surface roughness increases the electroactive surface area and promotes efficient electron transfer, while simultaneously reducing biofouling, a common limitation in long‐term biosensing applications.

### 4.4 Enhancing Electrode Kinetics

In glucose electrooxidation, slow electrode kinetics often result in delayed response times and incomplete oxidation of glucose, which affects sensitivity, linear detection range, and reproducibility. Incorporating conductive carbon‐based nanomaterials, such as rGO and multi‐walled CNTs, can significantly enhance electron transfer rates and shorten sensor response times. Their fibrous and mesoporous architecture also enables efficient mass transport and increased analyte loading capacity, both of which are critical for achieving fast, reliable signal output.^[^ ^84^, ^85^ ^]^ Response time is defined as the duration required for the sensor to reach 95% of its steady‐state current upon exposure to glucose. A fast response is critical for real‐time monitoring, where rapid fluctuations in glucose levels must be captured without delay. Most high‐performance glucose sensors achieve response times in the range of 1–10 s, which is largely facilitated using high surface‐area materials such as noble metal nanoparticles, metal oxides, and nanocomposites. These materials enable rapid electron transfer and efficient mass transport of glucose molecules at the electrode interface.^[^ ^63^ ^]^ Jia et al. engineered a glucose sensor by decorating dendritic‐like AuNSs with PtNPs on a GCE, aimed at boosting electrocatalytic oxidation of glucose.^[^ ^86^ ^]^ The synergistic effect of the high surface area of the dendritic AuNSs and the catalytic activity of PtNPs facilitated rapid electron transfer kinetics, resulting in a high sensitivity of 275.44 μA mM^−1^ cm^−2^ across a broad linear range of 0.1–14 mM and a fast response time of 2 s. The system also exhibited good reproducibility, making it promising for practical applications.

The design of nanostructured electrode surfaces continues to be a critical strategy for enhancing the performance of nonenzymatic glucose sensors. Guo et al. engineered gold surfaces with a lamellar ridge morphology, which significantly increased the electroactive surface area and improved catalytic kinetics.^[^ ^87^ ^]^ Compared to conventional flat gold‐modified electrodes, the lamellar ridge structures exhibited a 5.8‐fold increase in sensitivity and a 3.7‐fold reduction in the LOD for glucose, emphasizing the impact of surface topology on electrochemical performance. In a related effort, Zhong et al. developed a 3D nanoporous gold electrode for nonenzymatic glucose sensing. This architecture enabled rapid mass transport and provided a high density of active sites, resulting in excellent sensitivity, fast response time, and strong selectivity toward glucose in the presence of interfering species.^[^ ^88^ ^]^ The structural advantages of nanoporous‐Au highlight its promise for real‐time biosensing applications, particularly in CGM systems. Zhang et al. further expanded the material landscape by synthesizing Co‐MOFs and cobalt hydroxide structures via ionothermal and one‐pot conversion methods. These cobalt‐based materials demonstrated notable improvements in both sensitivity and response time, attributed to their high surface area, porous morphology, and favorable redox activity.^[^ ^89^ ^]^ The integration of transition metal‐based frameworks introduces new opportunities for designing robust, enzyme‐free sensing platforms that operate under physiological conditions.

The linear detection range represents the concentration interval over which the sensor maintains a directly proportional relationship between glucose concentration and current output. A broad and linear range enables accurate detection across clinically relevant thresholds, including hypoglycemic (<3.9 mM) and hyperglycemic (>7.8 mM) states. Electrode modification strategies have been employed to extend linearity. For example, performing measurements in hydroxide‐rich solutions facilitates the oxidation of glucose, enhancing both the range and response time.^[^ ^90^ ^]^ Distinctions between calibration range, dynamic range, and working range are also important. The calibration range is the interval validated through calibration, the dynamic range encompasses all detectable concentrations (though not necessarily linear), and the working range refers to the subinterval where semi‐linear behavior is acceptable for practical purposes.^[^ ^11^, ^91^, ^92^ ^]^ Dong et al. fabricated a high‐performance glucose sensor using a nanocomposite of α‐nickel hydroxide and rGO (α‐Ni(OH)_2_‐rGO) supported on a nickel foam substrate.^[^ ^93^ ^]^ The porous, conductive architecture provided ample electroactive sites and excellent electron mobility, allowing glucose detection in a wide concentration range from 0.5 mM to 22.5 mM. The use of nickel‐based materials offers a cost‐effective and stable alternative to noble metals, particularly for applications requiring long‐term deployment without enzyme degradation.

Accurate interpretation of low‐concentration glucose detection requires defining several key statistical limit metrics. The limit of blank (LOB) is the highest glucose concentration expected when analyzing a blank sample and represents the upper threshold of background noise. The DL refers to the lowest concentration that can be reliably distinguished from a blank sample with 99% confidence. The LOD is the minimal detectable concentration above the detection limit, with a false negative rate of less than 1%. Finally, the limit of quantification (LOQ) is the lowest concentration that can be measured with acceptable levels of accuracy and precision. For example, a glucose sensor with an LOD of 30 μM is capable of reliably detecting glucose at concentrations as low as 30 μM.^[^ ^94^ ^]^ These thresholds are pivotal for tailoring sensors for clinical or nonclinical settings, especially in wearable or minimally invasive applications where glucose fluctuations must be precisely tracked.^[^ ^95^, ^96^ ^]^

Alternatively, Hill‐Dick et al. presented a nanocoral reduced Ag (R‐Ag) based nonenzymatic glucose sensor with high sensitivity and low operating potential (0.1 V vs. Ag/AgCl), addressing key limitations of conventional Ag sensors.^[^ ^97^ ^]^ The R‐Ag sensor offers linear detection from 100 μM to 2.5 mM, with a low LOD of 1.78 μM and strong long‐term stability (≈45 days) and exhibited good selectivity despite common interferences. However, signal saturation at higher glucose concentrations (>20 mM) indicates surface limitations due to anion adsorption. This highlights the R‐Ag sensor's promise for reliable, low‐power glucose sensing. Mustafa et al. reported a composite sensor based on NiO and Co_3_O_4_ incorporated onto rGO (NiO‐Co_3_O_4_/rGO), which exhibited a LOD of 0.13 μM, attributed to the synergistic catalytic activity of the bimetallic oxide and the high surface area and conductivity of rGO.^[^ ^98^ ^]^ Additionally, Huo et al. developed a nanocomposite electrode by modifying CNTs with AuNPs and zirconia (AuNPs/ZrO_2_/CNT), achieving a LOD of 0.95 μM.^[^ ^99^ ^]^ The inclusion of zirconia not only improved structural stability but also enhanced antifouling properties, while AuNPs contributed to enhanced electrocatalytic activity and electron transfer efficiency.

These parameters form a comprehensive assessment of electrochemical glucose sensor performance. Devices optimized across these dimensions are more likely to be successfully translated into clinical, wearable, or implantable platforms where reliability, speed, and longevity are paramount. Beyond core analytical metrics such as sensitivity and detection limits, operational performance parameters like response time, reproducibility, and stability are essential for assessing a sensor's readiness for real‐world applications, particularly in dynamic or CGM systems.

### 4.5 Methodological Considerations for LOD Determination

LOD is a critical analytical performance parameter that defines the lowest concentration of glucose that can be reliably differentiated from background noise with a high degree of statistical confidence, typically set at the 99% confidence level.^[^ ^100^ ^]^

Accurately determining the LOD is essential for evaluating a sensor's applicability in physiological or trace‐level monitoring scenarios, especially where clinical relevance hinges on detecting glucose in micromolar concentrations or less.

#### 4.5.1 Signal‐to‐Noise Ratio (S/N) Method

Several methodologies have been established for calculating LOD, each with its strengths and appropriate use cases depending on the sensor architecture, experimental design, and data quality. The most widely accepted approaches are based on signal‐to‐noise ratio (S/N) and calibration curve‐based models. As summarized below, the S/N method is the most straightforward approach for estimating LOD, and it is based on the ratio of the sensor's analytical signal to the noise baseline. In this approach, the LOD is defined as the concentration that yields a signal equal to three times the standard deviation (*σ*) of the noise (background response)

(6) $$LOD=\frac{3\times \delta_{noise}\times C_{initial}}{Signal}$$

where *δ*_*noise*_ is the standard deviation of baseline noise, *C*_*initial*_ is the concentration of the lowest spiked glucose sample, and *Signal* is the measured response corresponding to *C*_*initial*_. This method is particularly useful for real‐time amperometric or chronoamperometric measurements where current fluctuations from blank samples (e.g., PBS without glucose) define the noise level.

#### 4.5.2 Calibration Curve‐Based Method

**Figure 10:**
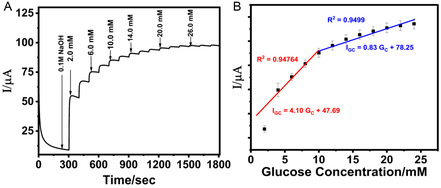
A) Chronoamperometric response by LIG/ZnO/Pd electrode towards glucose varying glucose concentration and B) the corresponding current versus glucose concentration.^[^ ^101^ ^]^ Adapted with copyright permission from MDPI.

The most rigorous and statistically grounded approach involves fitting a linear calibration curve of current (or current density) versus glucose concentration (**Figure** **10**).^[^ ^101^ ^]^ The slope of the curve (*S*) represents the sensitivity of the sensor, and the LOD is calculated as

(7) $$\mathrm{LOD}=\frac{3\times \delta_{blank}}{S}$$

or alternatively

(8) $$\mathrm{LOD}=\frac{3.3\times \delta_{y}}{S}$$

where $\delta_{blank}$ is the standard deviation of repeated blank measurements, $\delta_{y}$ is standard deviation of sensor responses for multiple measurements at low glucose concentrations, and *S* (sensitivity) slope of the calibration curve (μA mM^−1^ cm^−2^). These equations provide a robust statistical framework for evaluating sensitivity and detection limits based on real data and are especially applicable when the calibration exhibits strong linearity (R^2^ > 0.99).

**Figure 11:**
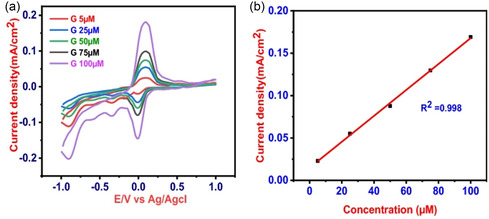
A) CV response of CNT/CuO towards different glucose concentrations at a scan rate of 10 mV s−1. B) Calibration curve in the linear range of 5–100 μM.^[^ ^48^ ^]^ Adapted with copyright permission from Springer.

**Figure** **11** illustrates a typical CV voltammogram and the corresponding calibration plot for a CNT/CuO composite electrode operating across a glucose concentration range of 5–100 μM. In this scenario, the LOD can be accurately calculated from the slope of the linear regression and the standard deviation of the blank, ensuring methodological transparency and reproducibility.

#### 4.5.3 Logarithmic Calibration Model

**Figure 12:**
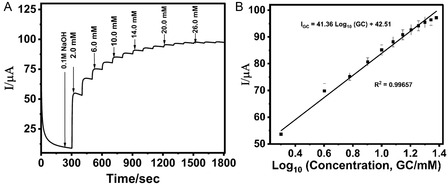
A) Chronoamperometric response by LIG/ZnO/Pd electrode towards glucose varying glucose concentration and B) the corresponding current versus Log_10_ of glucose concentration.^[^ ^101^ ^]^ Adapted with copyright permission from MDPI.

In certain instances, calibration is performed using a semi‐logarithmic relationship by plotting current against the log_10_ of glucose concentration (**Figure** **12**A–B). This can be useful for sensors with nonlinear or sigmoidal responses. The LOD is calculated as

(9) $$\mathrm{LOD}=10\frac{3\times \delta_{blank}-I_{y}}{S}$$

where $\delta_{blank}$ is the standard deviation of the blank, $I_{y}$ is the y‐intercept of the logarithmic calibration plot and *S* is the slope of the semi‐log curve. This formulation is less commonly used but is applicable to systems exhibiting logarithmic sensitivity in lower concentration regimes.

#### 4.5.4 Blank‐Only Statistical Method

In cases where spiking with glucose is not immediately feasible, the LOD can be estimated solely from blank signal measurements, assuming a Gaussian distribution of baseline responses

(10) $$\text{LOD}=\mu_{blank}+3\delta_{blank}$$

where $\mu_{blank}$ is the mean of the blank and $\delta_{blank}$ is the standard deviation of the blank.

Although this method is more conservative and less robust in the absence of an actual analyte, it offers a preliminary estimate of sensor detection capability under noise‐limited conditions.

#### 4.5.5 LOB‐Derived LOD

An advanced statistical model introduces the concept of the LOB, which is defined as the highest signal expected in the absence of analyte. The LOD is then computed using

(11) $$\text{LOD}=\text{LOB}+{1.65\delta }_{blank}$$

where $\delta_{blank}$ is the standard deviation of the blank. This approach distinguishes between baseline fluctuations (LOB) and actual analyte‐induced responses, increasing confidence in detection threshold determination, particularly in clinical diagnostics where false positives must be minimized.^[^ ^102^ ^]^

#### 4.5.6 Calibration Linearity and Interpretation

Accurate LOD determination is contingent upon a well‐fitted calibration curve. For amperometric sensors, linearity is usually assessed across multiple glucose concentrations, with repeated measurements (n ≥ 3) at each level to calculate average current response and standard deviation. R^2^ values ≥ 0.99 indicate excellent linearity, while residuals and heteroscedasticity analysis can be used to assess deviations from ideal behavior. Outliers or sensor drift must be addressed before calculating the slope and intercept to ensure the LOD reflects genuine performance rather than artefactual results. Furthermore, electrodes exhibiting high background current or unstable baselines are typically be excluded from LOD estimation unless corrected through baseline normalization or differential techniques.

Moreover, precise LOD estimation provides a foundational parameter for benchmarking sensor performance against clinical and regulatory requirements. While multiple methods exist, calibration curve‐based approaches coupled with rigorous noise analysis remain the gold standard. Ultimately, the reported LOD is interpreted in conjunction with linearity, reproducibility, and selectivity to comprehensively validate sensor utility for real‐world diagnostic or wearable applications.

## 5 Emerging Trends in Noninvasive Electrochemical Glucose Monitoring

Following the ongoing efforts to overcome challenges in sensitivity, selectivity, biocompatibility, and stability in invasive electrochemical glucose sensors, a significant research frontier has emerged: noninvasive and minimally invasive glucose monitoring. These approaches aim to eliminate the discomfort, infection risks, and compliance issues associated with frequent blood sampling, while offering real‐time, continuous monitoring for better glycemic control. However, these systems face their own set of challenges, particularly in ensuring accuracy and physiological relevance across diverse biological matrices.

### 5.1 Electrochemical Detection in Alternative Biofluids

Emerging technologies aim to overcome long‐standing limitations related to sensor accuracy, signal stability, and physiological variability. Noninvasive glucose monitoring via electrochemical sensing in alternative biofluids, including sweat, saliva, tears, and urine, has been widely investigated. These matrices offer accessible, nonblood‐based indicators of glucose levels, albeit at significantly lower concentrations (e.g., micromolar versus millimolar in blood), demanding ultrasensitive sensor platforms. These approaches enable noninvasive sampling but introduce new challenges related to matrix variability, lower analyte concentrations, and cross reactivity. Sweat‐based sensors are among the most widely researched, often integrated into flexible patches or smart textiles. Devices using functionalized nanomaterials (e.g., graphene, CNTs) have shown linear glucose detection down to 10 μM. However, variability in sweat rate, pH, and analyte dilution and contamination from the skin surface pose reproducibility challenges and remain major barriers.

Geetha et al. reported a high‐precision nonenzymatic electrochemical glucose sensor based on a CNTs/CuO composite. The sensor exhibited a rapid response time of 2 s and demonstrated high selectivity when tested in artificial sweat, highlighting its suitability for real‐time, noninvasive monitoring applications.^[^ ^48^ ^]^ Expanding on the theme of wearable biosensing materials, Myndrul et al. introduced a ZnO/MXene‐based glucose sensor engineered for skin‐attachable and flexible applications.^[^ ^103^ ^]^ This device exhibited a sensitivity of 29 μA mM^−1^ cm^−2^ over a linear range of 0.05 to 0.7 mM and achieved a low LOD of 17 μM. The MXene matrix not only contributed to excellent electrical conductivity and mechanical flexibility but also offered a large surface area for effective analyte interaction. Li et al. further advanced MXene‐based biosensing by hybridizing Pt nanoparticles onto Ti_3_C_2_T_x_ MXene nanosheets (Pt/MXene), yielding a broad linear detection range up to 8 mM under physiologically relevant neutral pH conditions for sweat based sensing.^[^ ^104^ ^]^ The combination of noble metal electrocatalysis with MXene's tunable surface chemistry resulted in improved glucose oxidation kinetics and device stability. In a complementary approach, Huang et al. developed a skin‐attachable, self‐powered epidermal glucose biosensor integrated into a PDMS substrate. The device converted biochemical signals from sweat into electrical outputs with a sensitivity of 0.11 mV mM^−1^, offering a promising foundation for battery‐free wearable glucose monitoring systems.^[^ ^105^ ^]^ Similarly, Banerjee and Slaughter reported a glucose‐powered abiotic biofuel cell using AuNP ink‐printed electrodes on bacterial nanocellulose with a co‐Pt anode and Ag_2_O‐MWCNT cathode.^[^ ^5^ ^]^ The cell achieved a peak power density of 0.087 mW/cm^2^ at 0.35 V (20 mM glucose) and showed linear performance in serum. It generated sufficient power to light an LED, demonstrating promise for low‐power wearable applications. Further pushing the frontier of wearable sensing, Zhu et al. developed a novel electrocatalytic system for nonenzymatic glucose oxidation.^[^ ^106^ ^]^ The wearable platform incorporated PdNPs encapsulated in cobalt‐based zeolitic imidazolate frameworks (ZIF‐67), functioning as an efficient electrocatalyst for water‐splitting‐driven glucose detection. This architecture facilitated continuous, low‐power monitoring by integrating catalytic water oxidation with electrochemical glucose sensing, presenting an innovative avenue for self‐powered and skin‐conformal sensors.

Saliva and tear‐based glucose sensors, often in the form of smart contact lenses or oral patches, offer attractive platforms for continuous monitoring. Recent prototypes have employed microfluidic integration and wireless telemetry for real‐time glucose tracking. Tear glucose sensors, particularly those embedded in smart contact lenses, are promising for continuous monitoring with minimal user burden. Saliva‐based detection offers a nonintrusive route but faces issues with high protein content and fluctuating glucose levels influenced by dietary intake and salivary flow rate. Current designs leverage nanocomposite films and signal amplification strategies to improve specificity and sensitivity in this matrix.

Yang et al. developed a 3D ordered CuO nanoflake array designed for miniaturized electrochemical glucose sensing in saliva.^[^ ^107^ ^]^ The sensor exhibited a high sensitivity of 4954 μA mM^−1^ cm^−2^ within a detection range of 1.0 to 6.0 mM and demonstrated a low LOD of 0.1 μM. Its architecture contributed to long‐term operational stability, making it a promising candidate for POC diagnostics. Wang et al. further explored copper‐based nanocomposites by constructing a CuO‐SnO_x_ hybrid for continuous salivary glucose detection. This device achieved a sensitivity of 2303 μA mM^−1^ cm^−2^ across a broad linear range (1 μM to 6 mM), with a LOD of 0.1 μM, demonstrating improved electrochemical activity through synergistic effects between metal oxides.^[^ ^108^ ^]^

Expanding the approach to wearable biosystems, Chen et al. designed an ultrathin, flexible skin‐like biosensor integrated with a paper battery‐powered electrochemical dual‐channel configuration.^[^ ^109^ ^]^ Targeting oral fluid analysis, the system demonstrated a sensitivity of 130.04 μA mM^−1^, reflecting the growing potential of self‐contained, disposable, and autonomous biosensing platforms. Urine‐based detection has also gained momentum. Janmee et al. engineered a smartphone‐assisted, SPE modified with a CuO/ionic liquid/rGO nanocomposite.^[^ ^110^ ^]^ The device enabled single‐step glucose detection in human urine with a linear range of 0.03 to 7.0 mM, LOD of 0.14 μM, and a response time of 20 s. The integration with smartphone technology supports the move toward decentralized diagnostics and telemedicine applications. Moreover, Sempionatto et al. demonstrated an innovative wearable biosensing platform embedded within eyeglass frames, incorporating wireless electronics for the simultaneous monitoring of glucose and other biomarkers, including alcohol and vitamins.^[^ ^111^ ^]^ This multifunctional system emphasizes the feasibility of multiplexed, real‐time health monitoring through ergonomically integrated sensors.

### 5.2 Real Sample Detection

Real sample detection serves as a critical benchmark for evaluating the translational potential and practical applicability of electrochemical glucose sensors. Unlike idealized laboratory conditions, real biological and environmental samples contain complex matrices with multiple interfering species, variable pH, and diverse ionic compositions. Therefore, the ability of a sensor to accurately and reproducibly detect glucose in such conditions is essential for validating its performance for clinical diagnostics, wearable health monitoring, and industrial quality control. While much of the sensor validation in prior sections involved synthetic matrices simulating fluids such as blood, sweat, saliva, urine, tears, and interstitial fluid, true real‐sample testing introduces greater biological variability. This includes patient‐derived samples or nonbiological systems such as fermented products and agricultural extracts where glucose concentration monitoring is relevant for bioprocessing and food safety applications.

#### 5.2.1 Quantitative Metrics and Recovery Rate

A standard metric used to assess performance in real sample detection is the recovery rate, which quantifies the accuracy of glucose concentration measurement when spiked samples are used. The recovery rate is calculated using the following equation

(12) $$\text{Recovery}\;\text{Rate}=\left(\frac{C_{found}-C_{original}}{C_{added}}\right)\times 100\%$$

where *C*_*found*_ is the measured concentration in spiked sample, *C*_*original*_ is the concentration in unspiked sample, and *C*_*added*_ is the concentration of the spiked glucose. High recovery rates (>95%) indicate strong accuracy and low matrix interference, validating the sensor's clinical or industrial feasibility. Sensors achieving consistent recovery across a broad range of analyte concentrations and fluid types are more likely to succeed in POC settings, especially for CGM where real‐time precision is critical.

#### 5.2.2 Human Biological Fluids

Kihal et al. demonstrated real blood glucose detection using a facile electrodeposition of nickel hydroxide onto a 3D Inconel 625 foam substrate.^[^ ^112^ ^]^ The sensor exhibited high sensitivity, low interference (<4%), and excellent recovery rates ranging from 93.27% to 103.60%, validating its potential for whole‐blood applications. The Inconel‐based scaffold provided mechanical robustness, while the Ni(OH)_2_ layer facilitated efficient glucose oxidation in alkaline media, ensuring sensor stability in a complex redox environment. Similarly, Pavadai et al. developed a nickel‐based electrode integrated into a MOF for glucose sensing in sweat and saliva.^[^ ^113^ ^]^ Operating in NaOH electrolyte, the sensor achieved recovery rates of 96.4–98.1% in sweat and 90.0–98.5% in saliva. These results highlight the electrode's capability to maintain sensitivity and reproducibility across biofluids with low glucose concentrations and high variability in ionic content. Importantly, sweat and saliva present promising noninvasive sampling options for CGM, and such performance benchmarks highlight the practicality of MOF‐based platforms in wearable devices.

Cui et al. fabricated a hybrid sensing electrode by combining laser‐induced graphene (LIG) with MXenes and Au nanoparticles onto CuO surfaces.^[^ ^114^ ^]^ The sensor enabled glucose detection in urine samples up to 5 mM, achieving recovery rates between 96.8% and 102.95%. The integration of conductive MXene sheets with nanostructured CuO offered a synergistic effect on electron transfer and signal amplification, while the LIG framework enhanced flexibility and miniaturization potential. Given the moderate glucose concentrations in urine and the variable matrix composition, this recovery values demonstrate high sensor resilience under real‐use conditions.

#### 5.2.3 Nonbiological Applications

Beyond physiological detection, real sample glucose monitoring has applications in industrial fermentation processes, agricultural systems (e.g., monitoring plant sap or soil eluates), and processed food labeling. While limited studies address these domains in the current literature, extending electrochemical sensor validation to such matrices is increasingly important for environmental monitoring and food traceability. For example, future sensors must contend with organic contaminants, microbial biofilms, and variable osmolarity, which present different interference and stability challenges compared to biological fluids.

The successful deployment of glucose sensors in real samples represents a key milestone in the development lifecycle. These nonenzymatic glucose sensors herein demonstrate that carefully engineered material platforms, particularly those combining transition metal oxides, MOFs, carbon‐based frameworks, and polymeric coatings enable high‐accuracy, low‐interference detection across a range of real‐world scenarios.

### 5.3 Reverse Iontophoresis and Minimally Invasive Microneedles

Reverse iontophoresis is a minimally invasive technique that extracts interstitial fluid through the skin using a mild electrical current was previously commercialized and is now discontinued, GlucoWatch. Though promising, issues with skin irritation and inconsistent extraction efficiency limited its utility. Next‐generation platforms combine transdermal techniques with nanostructured electrodes, biocompatible hydrogels, and closed‐loop feedback systems to enable better glucose recovery and real‐time monitoring. Zhu et al. explored the influence of pH on the transdermal extraction efficiency of glucose flux, a crucial parameter for calibrating glucose concentrations in noninvasive monitoring systems.^[^ ^115^ ^]^ Through a combination of experimental and theoretical analyses, the study demonstrated that subcutaneous glucose levels of 5 mM and 10 mM correlated with transdermal glucose concentrations of 0.08212 mM and 0.14639 mM, respectively, per unit change in pH. These findings stress the importance of real‐time pH compensation algorithms for improving the accuracy of CGMs relying on skin‐based sampling. Addressing challenges in drug delivery and biomarker extraction through the skin, Moore et al. investigated the use of reverse iontophoresis to quantify the bioavailability of topically applied compounds across the stratum corneum.^[^ ^116^ ^]^ This study not only provided insights into the ionic permeability of the skin barrier but also highlighted the technique's potential in glucose monitoring by facilitating the controlled extraction of interstitial fluid for analysis.

Microneedle‐based arrays represent a hybrid solution between invasive and noninvasive methods. These devices penetrate the skin's stratum corneum without reaching nerve endings, making them pain‐free yet effective in accessing ISF. Functionalized microneedles made from biodegradable polymers, metallic nanocomposites, or hydrogels can serve as platforms for electrochemical or colorimetric glucose detection. When integrated with wireless electronics, these sensors allow real‐time, minimally invasive monitoring with improved user compliance. Further, these platforms often integrate biosensors into biodegradable or dissolvable microneedle patches, enabling CGM with minimal discomfort. Ribet et al. demonstrated a proof‐of‐concept CGM system leveraging the structural and functional benefits of microneedles.^[^ ^117^ ^]^ Their system integrated a miniaturized electrochemical sensing cell within the lumen of a hollow silicon microneedle. This configuration enabled localized ISF sampling with significantly reduced tissue trauma and improved measurement accuracy. Comparative analysis with commercial CGM platforms indicated promising accuracy and potential for miniaturized, low‐pain alternatives to traditional subcutaneous sensors.

Complementing these advancements, Abdullah et al. provided a detailed review of diverse microneedle technologies, emphasizing operational mechanisms, material selection, and the clinical advantages of ISF extraction.^[^ ^118^ ^]^ Their work categorized microneedle systems into solid, coated, dissolving, and hollow types, emphasizing their role in enhancing real‐time, minimally invasive biochemical analysis.^[^ ^119^ ^]^ Further supporting the feasibility of large‐volume ISF sampling, Miller et al. reported a hollow microneedle array capable of extracting 20–60 μL of dermal ISF from both human and rat models without causing blistering or tissue damage. This study marks a significant milestone for biosensor calibration and diagnostic testing, where sufficient analyte volumes are often a critical bottleneck.^[^ ^120^ ^]^

### 5.4 Integration with Wearable and IoT‐Enabled Systems

The convergence of wearable electronics, wireless communication, and cloud‐based data processing has fueled the development of smart glucose‐monitoring systems and IoT platforms for personalized health monitoring tools. Noninvasive sensors are now integrated into smartwatches, skin patches, and textiles, allowing real‐time data collection, analysis, and transmission to smartphones or remote clinicians. Wearables incorporating Bluetooth Low Energy (BLE), NFC, or RFID modules enable seamless health data logging and patient‐specific alerts. Combined with AI‐driven data analytics, these systems can generate glucose trends, detect hypoglycemic or hyperglycemic events, and deliver timely alerts to users or healthcare providers, thereby offering a pathway to personalized diabetes management and preventive care. Furthermore, cloud‐based platforms allow for remote patient monitoring, telemedicine integration, and decision support, making these systems attractive for both clinical and at‐home applications. For instance, Abubeker et al. proposed an IoT glucose monitoring (iGM) based on ESP32 module with organic LED (OLED), open‐source software with embedded C structure and Amazon Web Services IoT core infrastructure to transform diabetes care and improve overall quality of human life. The accuracy was 98.82 and 98.04% after 10 h of fasting and after 2 h of breakfast respectively.^[^ ^121^ ^]^

Huang et al. provided a comprehensive review highlighting the integration of next‐generation CGMs with wearable technologies and AI‐driven closed‐loop insulin delivery systems.^[^ ^122^ ^]^ Their analysis emphasized the role of machine learning algorithms in enhancing glucose prediction accuracy, improving sensor calibration, and enabling adaptive insulin dosing, which collectively reduce the physiological burden of diabetes management. The review also discussed regulatory, cybersecurity, and interoperability challenges that must be addressed to facilitate clinical deployment. Additionally, Ahmed et al. conducted a systematic review evaluating the efficacy of wearable technologies in noninvasive blood glucose prediction.^[^ ^123^ ^]^ Their assessment factored in methodological quality and technological readiness, emphasizing that wearable‐based models, particularly those incorporating photoplethysmography (PPG) and near‐infrared (NIR) spectroscopy, show promise in mitigating the invasiveness of current CGM systems. Wrist‐worn devices were identified as the most adopted form factor, largely due to user comfort, accessibility, and compatibility with daily life routines.^[^ ^124^ ^]^ Together, these studies illustrate a clear trajectory toward intelligent, user‐centered diabetes monitoring systems.

## 6 Broad Applications and Future Outlook of Glucose Sensing Technologies

Glucose sensing technologies have demonstrated substantial versatility, with impactful applications across diverse fields including clinical diagnostics, biotechnology, environmental monitoring, food quality control, and wearable electronics. The evolution of glucose sensors from invasive diagnostic tools to intelligent, minimally invasive and noninvasive platforms continues to drive innovation in both healthcare and industrial sectors. In clinical and personal healthcare, glucose sensors serve as a cornerstone in diabetes management, enabling real‐time monitoring of blood glucose levels. CGM systems represent a transformative approach, offering dynamic, noninvasive glucose tracking that improves patient compliance, minimizes hypoglycemic events, and enhances treatment personalization.^[^ ^63^, ^123^, ^124^ ^]^ In hospital settings, accurate glucose sensing is essential for perioperative glycemic control and intensive care monitoring, where fluctuations in glucose levels are linked to patient outcomes. Miniaturized portable glucometers further empower patients to manage their health outside clinical environments. These devices combine electrochemical detection with user‐friendly interfaces and connectivity features, facilitating at‐home monitoring and remote physician access.

Hassan et al. provided a comprehensive review on the mechanistic evolution of electrochemical glucose sensors, noting that recent advances in nanomaterials, sensor design, and digital health frameworks offer the potential to transition from static sensing devices to fully integrated systems capable of real‐time physiological monitoring and predictive analytics.^[^ ^125^ ^]^ These systems could enable continuous health status tracking, remote physician oversight, and early intervention strategies for comorbid conditions such as hypertension and cardiovascular risk. Building on this vision, Alam et al. reported on an IoT‐based system designed for the seamless collection and transmission of patient‐specific diabetic data. Their noninvasive monitoring approach emphasized patient comfort while enabling longitudinal data collection and physician access to real‐time glucose trends, thereby supporting clinical decision‐making and personalized treatment planning.^[^ ^126^ ^]^ Furthermore, Karpova et al. advanced the concept of continuous sweat‐based diagnostics through wearable biosensors designed for simultaneous monitoring of glucose and hypoxia.^[^ ^127^ ^]^ Their findings highlight the feasibility of real‐time metabolic profiling via noninvasive biofluids, paving the way for multi‐analyte monitoring platforms tailored for chronic disease management.

The integration of glucose sensors into wearable devices such as smartwatches, fitness bands, and skin patches represents a frontier in personalized health and real‐time metabolic monitoring. These platforms offer minimally invasive or noninvasive glucose tracking via alternative biofluids (e.g., sweat, saliva, or interstitial fluid), granting users deeper insights into their glucose dynamics throughout daily activities.^[^ ^128^ ^]^ Wearable biosensors hold promise for preventive care, early disease detection, and lifestyle optimization, though clinical accuracy and regulatory validation remain ongoing challenges. For instance, Ngo et al. proposed an IoT‐based smart textile system capable of monitoring multiple physiological parameters, including body temperature, heart rate, step count, and sleep cycles.^[^ ^129^ ^]^ While this integrated wearable system shows promise for real‐time health surveillance, it remains at a nascent stage of development and is currently limited by short battery life and insufficient clinical validation, highlighting the need for further optimization and in vivo testing. In parallel, Bent et al. demonstrated a wearable prototype with noninvasive capabilities for glucose and HbA1c monitoring.^[^ ^130^ ^]^ Their findings indicate a strong correlation with traditional CGM systems, suggesting that such devices could eventually serve as reliable alternatives for glycemic control if long‐term stability and calibration issues are adequately addressed. Moreover, Diez et al. explored the integration of flash glucose monitoring systems and digital disease management technologies through smartwatches and smartphone applications.^[^ ^131^ ^]^ Although their work suggests the potential for real‐time, user‐friendly diabetes management interfaces, it lacks depth in terms of technical performance metrics and user outcomes, presenting a clear opportunity for future investigation.

In biotechnology and pharmaceutical research, glucose sensors play a vital role in monitoring cell metabolism, assessing fermentation kinetics, and optimizing bioprocess parameters. Real‐time glucose tracking supports optimal growth conditions in microbial and mammalian cell cultures, where nutrient depletion or imbalance can hinder productivity. In drug development, sensors aid in evaluating the metabolic response to therapeutic compounds, particularly those targeting glucose homeostasis in metabolic disorders such as diabetes, obesity, and cancer.^[^ ^132^ ^]^ Elsheakh et al. developed an innovative blood glucose monitoring biosensor utilizing a multiband split‐ring resonator monopole antenna, achieving a sensitivity of 24 MHz/mM.^[^ ^133^ ^]^ This sensor demonstrates significant potential for applications in pharmaceutical research, particularly in the precise monitoring of glucose levels. Complementing this, Ding et al. investigated the integration of glucose sensors within feedback control systems to dynamically monitor glucose uptake and overall metabolic activity in Escherichia coli and its various strains, highlighting the utility of biosensors in real‐time cellular metabolism analysis.^[^ ^134^ ^]^

In the food and beverage industry, glucose sensors are employed for nutritional analysis, quality control, and fermentation monitoring, ensuring product consistency and regulatory compliance.^[^ ^135^, ^136^ ^]^ Accurate glucose detection informs caloric labeling and helps prevent spoilage during production and storage. In environmental monitoring, glucose‐based biosensors are instrumental in assessing biochemical oxygen demand as an indirect measure of organic load in wastewater. This application is critical for evaluating the efficiency of wastewater treatment processes and ensuring regulatory standards are met.^[^ ^137^ ^]^ Li et al. explored the potential of Amplex Red as both an indicator and mediator in rapid and straightforward colorimetric and electrochemical glucose sensing platforms.^[^ ^138^ ^]^ Their findings suggest that such sensors can be effectively adapted for applications in food quality control and beverage monitoring, providing a promising approach for ensuring product safety and freshness. Addressing the broader context of food spoilage and freshness monitoring, Kuswandi et al. discussed the integration of chemical and biosensors within intelligent food packaging systems.^[^ ^139^ ^]^ These systems enable real‐time monitoring of critical parameters such as CO_2_ or O_2_ concentration, pH, and temperature, facilitating dynamic assessment of food quality through smart labeling technologies. Beyond food applications, glucose sensors have also been applied in environmental monitoring, particularly wastewater management. For instance, Jasim et al. developed a ZnO/CuO‐based sensor capable of detecting glucose and other organic compounds, demonstrating its potential utility in real‐time industrial wastewater effluent quality monitoring.^[^ ^140^ ^]^ Complementing this, Kesari et al. provided a comprehensive review on the use of glucose and related sensors in wastewater treatment processes, highlighting their role in tracking biodegradable organic molecules.^[^ ^141^ ^]^ Such monitoring not only aids in ensuring effective wastewater treatment but also opens avenues for the safe reuse of treated water in agricultural and industrial contexts.

### 6.1 Future Outlook

Glucose sensing technologies have undergone a rapid and transformative evolution, catalyzed by the growing global demand for accurate, real‐time, and user‐centric monitoring tools, particularly in the management of diabetes. Key innovations have been driven by the pursuit of greater selectivity, sensitivity, and user convenience, resulting in the development of nonenzymatic electrochemical glucose sensors with increasingly sophisticated performance characteristics. Extensive research has focused on the development of nonenzymatic platforms utilizing advanced nanomaterials. Specifically, 2D and 3D nanostructured materials have shown great promise due to their high surface area, superior electrocatalytic activity, and efficient electron transfer kinetics.^[^ ^142^ ^]^ These materials have enabled the construction of nonenzymatic sensors with selectivity and sensitivity levels approaching those of enzymatic systems. Moreover, the tunability of surface functionalities has improved responsiveness to glucose and minimized interference from coexisting biomolecules, enhancing performance in complex biological environments.

Despite these advancements, several persistent challenges continue to hinder the widespread adoption of next‐generation glucose sensors, particularly in noninvasive, wearable, and long‐term monitoring applications. One of the most pressing issues is signal instability in complex biofluids such as sweat, saliva, interstitial fluid, and tears.^[^ ^143^ ^]^ These fluids exhibit significant compositional variability, including pH shifts, ionic strength fluctuations, and the presence of coexisting electroactive species, which can contribute to baseline drift, signal suppression, and false‐positive readings.^[^ ^144^ ^]^ Furthermore, the glucose concentrations in these matrices are typically an order of magnitude lower than in blood, increasing the requirement for ultra‐sensitive detection and robust interference rejection. To address this, future sensor designs must incorporate smart nanostructures that can selectively preconcentrate glucose, suppress nonspecific binding, and maintain catalytic activity in dynamic microenvironments. Emerging solutions include the integration of hydrogel‐nanomaterial composites, MOF‐based molecular sieves, and stimuli‐responsive polymer coatings to filter and stabilize analyte access while mitigating biofouling.

A second key challenge is calibration drift and reproducibility, especially in wearable and POC formats. Factors such as perspiration rate, ambient temperature, mechanical deformation, and skin‐electrode impedance variation can lead to unpredictable changes in sensor output. Conventional one‐time calibration approaches are insufficient under such conditions. Instead, adaptive multi‐point calibration algorithms, powered by machine learning, could be implemented to dynamically adjust for physiological and environmental fluctuations. Integration with secondary sensors for pH, hydration, or sweat rate could provide contextual data that enhances calibration precision through data fusion models. These intelligent systems will be critical to achieving clinically actionable glucose readouts, particularly in decentralized or resource‐limited settings.

From a materials perspective, a deeper understanding of how morphological characteristics such as crystallinity, porosity, particle size distribution, and hierarchical architecture influence long‐term sensor performance is needed. Many nanomaterials show excellent initial sensitivity but degrade rapidly due to oxidation, leaching, or structural collapse under continuous operation. Research efforts should focus on the design of mechanically robust, corrosion‐resistant nanocomposites, such as core‐shell structures or defect‐engineered carbons, that can withstand harsh biofluid environments while maintaining electrocatalytic activity. The synthesis of such materials must also prioritize scalability, cost‐efficiency, and batch‐to‐batch reproducibility to enable a pathway for commercial translation.

Additionally, integration with flexible and stretchable substrates, essential for wearable formats, presents unique fabrication and durability challenges. Issues related to delamination, cracking, and loss of conductivity during repeated mechanical deformation can impair sensor reliability. Emerging fabrication strategies, including laser‐induced graphene, inkjet printing, and direct‐write additive manufacturing, may offer pathways toward mechanically compliant yet highly conductive sensing platforms. Embedding sensing elements into textiles, skin patches, or microneedles is another promising route, with microneedle‐based sensors offering minimally invasive access to interstitial glucose without the discomfort associated with traditional blood sampling.

Another future development opportunity lies in the integration of sensors with wireless communication and real‐time analytics, supporting the convergence of glucose monitoring with broader digital health ecosystems. Coupling sensors with smartphone applications, Bluetooth‐enabled readouts, or cloud‐based dashboards not only improves user compliance and engagement but also enables longitudinal data tracking, remote clinician access, and behavioral feedback loops. The incorporation of artificial intelligence and predictive modeling into these platforms may further enhance disease management by forecasting glycemic trends, optimizing insulin dosing, or detecting anomalies in metabolic patterns.

The path to clinical adoption will depend not only on technical innovation but also on the standardization of validation protocols, biocompatibility testing, and regulatory approval pathways. There is an urgent need for harmonized standards governing analytical performance metrics (e.g., stability, accuracy under motion), biocompatibility, and safety in prolonged skin contact or subcutaneous deployment. Moreover, while invasive glucose sensors, such as finger‐prick blood tests and subcutaneous CGMs, continue to dominate clinical practice due to their accuracy, they pose concerns related to user discomfort, compliance, and infection risk. This has fueled growing interest in the development of noninvasive sensors capable of extracting glucose data from alternative matrices.^[^ ^145^ ^]^ The translation of such systems into reliable diagnostic tools requires resolving critical issues, including fluid‐specific calibration, improved correlation with blood glucose levels, and stable signal output under dynamic physiological conditions.

The convergence of biosensing platforms with wearable and mobile health technologies represents an exciting direction for the field.^[^ ^146^ ^]^ Integration into flexible substrates, coupled with wireless communication and smartphone‐based readouts, holds significant promise for improving user engagement, facilitating continuous monitoring, and enabling real‐time analytics. These technologies not only offer the potential to transform diabetes management but also support broader applications in preventive medicine and personalized healthcare. By linking dynamic glucose profiles with behavioral and therapeutic interventions, such platforms may empower patients and clinicians to make more informed decisions.

Looking forward, the development of reliable and accessible noninvasive glucose sensors will require interdisciplinary approaches that bridge nanomaterials science, biomedical engineering, artificial intelligence, and clinical research. Priority areas for innovation include the design of universal calibration algorithms capable of adapting to physiological and environmental variability, the enhancement of sensor performance in nonblood matrices, and the fabrication of scalable, biocompatible, and conformal sensor substrates suitable for prolonged wear. Just as critical is the establishment of standardized regulatory and validation frameworks to ensure safety, reproducibility, and clinical utility. In all, noninvasive glucose sensors represent a transformative opportunity to reshape diabetes diagnostics and chronic disease management.

Addressing current challenges such as selectivity in complex matrices, calibration drift, sensor longevity, and mechanical reliability requires a concerted, multidisciplinary effort. By advancing toward intelligent, adaptive, and user‐friendly sensor systems, the field is well‐positioned to transform glucose monitoring from an episodic diagnostic procedure into a seamless, continuous, and personalized health management tool.

## 7 Conclusion

Glucose sensing technologies have undergone significant evolution, advancing from early invasive electrochemical systems to increasingly sophisticated, miniaturized, and noninvasive platforms. Innovations in nanomaterials, surface engineering, and transducer design have enhanced sensor performance in terms of sensitivity, selectivity, and biocompatibility. The incorporation of materials such as rGO, CNTs, MOFs, and antifouling nanoparticles has addressed longstanding challenges associated with biofouling, signal drift, and matrix interference. These improvements have facilitated broader application across medical, biotechnological, environmental, and consumer health domains. Despite these strides, major obstacles remain, particularly in translating noninvasive and wearable glucose sensors from laboratory feasibility to reliable clinical use. The inherent complexity and variability of alternative biofluids, coupled with environmental influences and inter‐individual physiological differences, continue to affect accuracy and reproducibility. Furthermore, the lack of standardized calibration protocols, scalable manufacturing techniques, and clearly defined regulatory pathways has impeded widespread adoption and commercialization.

Future progress will depend on bridging materials, engineering, machine learning, and clinical research gap. Strategic priorities include the development of universal calibration algorithms, robust validation frameworks, and next‐generation substrates that are both flexible and biocompatible. Integrating these sensors with digital health tools and mobile diagnostics may unlock the full potential of personalized glucose monitoring, empowering users to manage metabolic health more effectively and proactively. As the field continues to innovate at the intersection of nanotechnology, biosensing, and wearable electronics, glucose sensors are poised to become not only more accurate and accessible but also transformative tools in the broader pursuit of real‐time, decentralized, and patient‐centered healthcare.

## Conflict of Interest

The authors declare no conflict of interest.
